# Using fibre photometry with a fluorescence resonance energy transfer-based biosensor to test efficacy of calpain inhibitors *in vivo*

**DOI:** 10.1093/braincomms/fcag150

**Published:** 2026-04-24

**Authors:** Katherine J Robinson, Anita J Turner, Holly I Ahel, Andrea Kuriakose, Anastasiya Potapenko, Maxinne Watchon, Stuart K Plenderleith, Nicholas A Everett, Simon McMullan, Angela S Laird

**Affiliations:** Motor Neuron Disease Research Centre, Macquarie Medical School, Faculty of Medicine, Health and Human Sciences, Macquarie University, Sydney 2109, Australia; Macquarie Medical School, Faculty of Medicine, Health and Human Sciences, Macquarie University, Sydney 2109, Australia; Motor Neuron Disease Research Centre, Macquarie Medical School, Faculty of Medicine, Health and Human Sciences, Macquarie University, Sydney 2109, Australia; Motor Neuron Disease Research Centre, Macquarie Medical School, Faculty of Medicine, Health and Human Sciences, Macquarie University, Sydney 2109, Australia; Motor Neuron Disease Research Centre, Macquarie Medical School, Faculty of Medicine, Health and Human Sciences, Macquarie University, Sydney 2109, Australia; Motor Neuron Disease Research Centre, Macquarie Medical School, Faculty of Medicine, Health and Human Sciences, Macquarie University, Sydney 2109, Australia; Motor Neuron Disease Research Centre, Macquarie Medical School, Faculty of Medicine, Health and Human Sciences, Macquarie University, Sydney 2109, Australia; Brain and Mind Centre, School of Psychology, Faculty of Science, The University of Sydney, Sydney 2006, Australia; Macquarie Medical School, Faculty of Medicine, Health and Human Sciences, Macquarie University, Sydney 2109, Australia; Motor Neuron Disease Research Centre, Macquarie Medical School, Faculty of Medicine, Health and Human Sciences, Macquarie University, Sydney 2109, Australia

**Keywords:** preclinical drug testing, drug optimization, calpain proteases, protease monitoring

## Abstract

Calcium-activated proteases, known as calpains, cleave proteins into smaller protein fragments, altering protein structure and function. Calpain overactivity is a pathological hallmark of many human diseases, including neurodegenerative diseases, which are characterized by excessive proteolytic cleavage and formation of protein aggregates. Current methods for determining calpain activity, such as examination of the presence or absence of calpain substrates in post-mortem tissue, are low-throughput, costly and retrospective, hampering the search for potentially therapeutically efficacious calpain inhibitor compounds. Here, we describe a novel methodology that allows real-time examination of calpain activity within the brain of the awake mouse. We used an adeno-associated viral (AAV) vector to express a fluorescence resonance energy transfer (FRET)-based calpain sensor in cerebellar neurons of male mice and measured real-time changes in FRET signal as a proxy for calpain activity via an implanted fibre optic cannula. This methodology was used to compare within-animal responses to different doses and administration routes of several calpain inhibitor compounds, including calpeptin, BLD-2736 and SNJ-1945. Using our AAV-calpain sensor and fibre photometry approach, we were able to obtain high-quality FRET recordings that were reproducible both between experimental animals and within experimental animals, allowing direct comparison of different calpain inhibitor compounds, enabling identification of the most efficacious treatment strategy that could be progressed further with preclinical treatment studies. As a positive control, we also tested changes in FRET signal in response to ionomycin, a known calcium ionophore. To our knowledge, this is the first report of real-time measurement of neuronal calpain activity *in vivo*. Future experiments could exploit this novel methodology to obtain calpain activity measurements alongside other pharmacological and behavioural/physiological measurements to increase the translational validity of preclinical models for drug development in diseases characterized by calpain overactivity.

## Teaser

Robinson *et al.* report a new methodology utilizing fibre photometry to detect changes in a fluorescence resonance energy transfer (FRET)-based calpain sensor, enabling measurement of neuronal calpain activity *in vivo*. This novel methodology enables direct comparison of the effectiveness of different calpain inhibitor compounds, doses and administration routes.

## Graphical Abstract

**Graphical Abstract:**
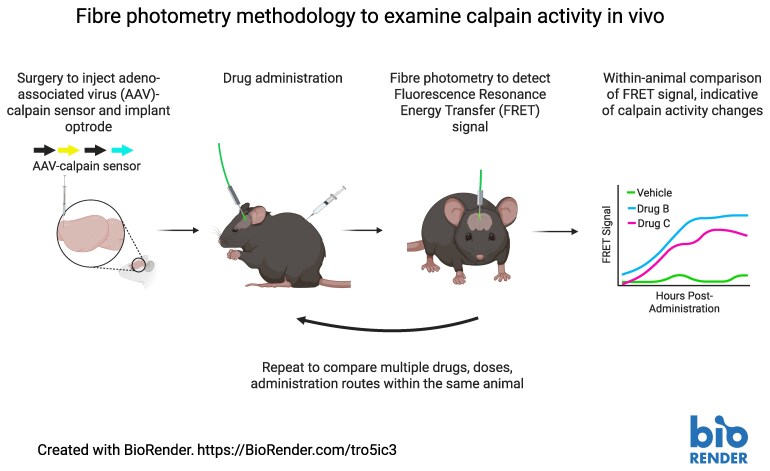

## Introduction

Proteolytic enzymes, known as proteases, selectively cleave peptide bonds, digesting full-length proteins into smaller fragments in an irreversible manner.^1^ Proteases are important for many biological processes, such as protein digestion, apoptosis, cell differentiation, cell signalling and immune responses.^2^ Proteolytic enzymes are known to modulate protein localization via removal of nuclear export or import sequences, regulate protein activity via the removal of functional sites, act as molecular messengers and contribute to processing cellular information.^1,3^ Altered protease activity is also a pathological signature of many diseases including diabetes,^4-6^ cancer,^7-10^ malaria,^11-13^ HIV,^14,15^ cardiovascular disease,^16-19^ pulmonary fibrosis,^20-22^ ischaemia,^23-25^ muscular dystrophy,^26-28^ traumatic brain injury^29-33^ and neurodegenerative diseases such as Alzheimer’s disease,^34-37^ Huntington’s disease,^38,39^ Parkinson’s disease,^40-42^ motor neuron disease^43-45^ and spinocerebellar ataxia type 3.^46-49^ Calpains, an intracellular cysteine protease, are highly evolutionarily conserved, with homologues detected in plants, fungi, invertebrates and mammals.^3^ In contrast to other proteases, calpains exert a modulatory effect on target substrates rather than destroying their target.^3,50^ Calpains can be ubiquitously expressed (calpain 1, 2, 5, 7, 10, 13, 15) or expressed exclusively in certain tissues (calpain 3, 6, 8, 9, 11, 12, 14 and 16).^50^ Calpain 1 and 2, the most extensively studied calpain isoforms, are highly expressed within the central nervous system and are activated by elevations in intracellular calcium concentrations.^3,50,51^ Many essential proteins are known to be cleaved by calpains, including cytoskeletal proteins α-spectrin, tau and α-synuclein, apoptosis-related proteins PARP1 and caspase-3 and autophagy-related proteins p62 and beclin-1.^3,50,52^ The activity of calpains is tightly regulated by calpastatin, the only known endogenous calpain inhibitor, in addition to changes in molecular conformation and neurotransmission, spatio-temporal expression patterns and calcium regulation.^3,51^

**Figure 1:**
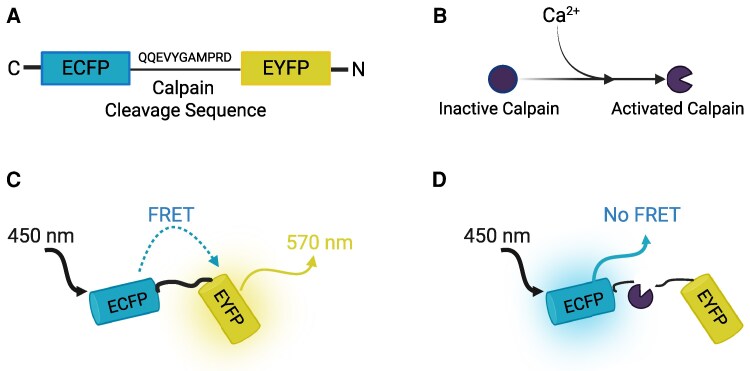
**Schematic diagram of calpain sensor construct and rationale.** (**A**) The amino acid sequence linking the ECFP and EYFP fluorophores. (**B**) Schematic depicting how changes in calcium signalling led to increased calpain activity. (**C**) Schematic display of how excitation of ECFP leads to fluorescence resonance energy transfer (FRET) and thus EYFP emission. (**D**) Activation of calpains can lead to cleavage of the sequence linking the ECFP and EYFP fluorophores, decreasing FRET and consequently leading to loss of EYFP emission. This figure was created in BioRender. Available at https://BioRender.com/efij9v3.

Historically, methods such as chromatography,^53-55^ immunohistochemistry,^56,57^ SDS-PAGE western blotting,^56,58,59^ mass spectrometry,^56^ 1D or 2D gel electrophoresis,^54,55,58^ PCR,^60^ casein zymographic assays^61^ and spectrometry assays involving radioactive labelling and trichloroacetic acid,^53,62,63^ have been used to examine the activity of calpains or degradation of calpain-substrates in brain tissue. However, these approaches cannot be used to detect calpain activity in living tissues. Findings from Boehm *et al.*^64^ suggest that the activity of calpains in stored post-mortem muscle tissue may be substantially decreased compared to calpain activity at time of death, thus examination of calpain activity in post-mortem tissue may not reflect physiological calpain activity,^65^ and there is a clear need to develop new tools to allow investigation of calpain activity within brain tissue *in vivo*, avoiding the potential confound of death-induced changes to calpain activity. To address this, we have developed a novel methodology that allows real-time examination of calpain activity within the brain *in vivo*. Our approach utilized a Förster resonance energy transfer (FRET)-based biosensor for the detection of calpain activity developed by Stockholm and colleagues,^66^ referred to throughout as the ‘calpain sensor’. Briefly, this sensor contains enhanced cyan fluorescent protein (ECFP) and enhanced yellow fluorescent protein (EYFP) separated by a short amino acid sequence (QQEVYGAMPRD) derived from the α-fodrin/α-spectrin protein, which is exclusively cleaved by calpain proteases and is not susceptible to cleavage by any other known proteases^66^ (Fig. 1A). Upon excitation of the ECFP fluorophore using ultraviolet (UV) illumination, ECFP will donate electrons to EYFP via FRET, leading to emission of EYFP fluorescence (Fig. 1B). However, an increase in calpain activation (Fig. 1C) and subsequent cleavage of the linking sequence leads to physical separation of EYFP and ECFP, preventing FRET (Fig. 1D). We utilized the calpain sensor in combination with fibre photometry, the same technology used for optical recording of calcium and neurotransmitter activity *in vivo,*^67^ to examine neuronal calpain activity in live animals.

The primary aim of this study was to develop a novel methodology that could aid and improve preclinical drug testing by comparing neuronal calpain activity following administration of a range of compounds known to modulate calpain activity. We have previously reported that treatment with calpain inhibitor compounds can produce therapeutic benefit in transgenic zebrafish modelling the fatal neurodegenerative disease spinocerebellar ataxia type 3 (SCA3, also known as Machado-Joseph disease).^68,69^ There is a wealth of evidence highlighting aberrant calpain activity in cellular and animal models of SCA3^46,49,70^ and SCA3 patient brain tissue,^71^ thus, we wanted to develop a methodology that would allow direct comparison of multiple calpain inhibitor compounds (including comparison of compound dose and administration route) in the same experimental animal; in this case, transgenic CMVMJD135 mice modelling SCA3 and wildtype littermates. This approach would allow identification of the most efficacious treatment strategy that can be pursued in future chronic treatment studies while minimizing the ethical and financial impact of the research.

In this study, we drove calpain sensor expression using an adeno-associated viral vector (serotype 1), targeting cerebellar neurons, a neuronal population that is affected in SCA3. We then implanted a fibre optic cannula and used a custom fibre photometry apparatus to record real-time changes in FRET signal, a proxy for calpain activity, following administration of different calpain inhibitor compounds. This novel methodology enabled within-animal comparison of different calpain inhibitor compounds, doses and administration routes, allowing confirmation that compounds are truly crossing the blood–brain barrier (BBB) and decreasing calpain activity within the cerebellum.

## Materials and methods

### *In silico* analysis of proteases predicted to cleave the calpain sensor linking sequence

To confirm the validity and specificity of the calpain sensor construct (Addgene 36182, gift from Isabelle Richard) previously developed by Stockholm *et al.*^66^ and Bartoli *et al.*,^72^ we used the recently developed platform ProsperousPlus,^73^ to generate protease cleavage predictions for the amino acid sequence (QQEVYGAMPRD) linking fluorophores within the calpain sensor. We examined the cleavage prediction scores of proteases such as calpains (calpain 1 and 2), caspases (isoforms 1–3, 6–8) and granzyme B.

### Cell culture validation of calpain sensor construct

Murine neuroblastoma × spinal cord hybrid (NSC-34) cells were grown under sterile conditions and maintained at 37°C in a 5% CO_2_ sterile incubator. Cells were grown in Dulbecco’s modified Eagle medium (DMEM) and supplemented with 10% foetal bovine serum (FBS). Cells were seeded into six-well plates and grown for 1–3 days until ∼90% confluent. Cells were transfected with 4 μL of Lipofectamine 2000 (Thermo Fisher Scientific), 2 μg of calpain sensor DNA and/or 2 μg of p3xFlag-CAPN1 (a gift from Yi Zhang, Addgene plasmid #60941) to overexpress calpain 1.^74^ Untransfected controls were treated with a solution of OptiMEM and Lipofectamine 2000 in the absence of any DNA. Transfections were performed in media containing 1% reduced serum OptiMEM and 10% FBS diluted in DMEM. ∼24 h after transfection, cell culture media was aspirated and replaced with OptiMEM. Cells were pre-treated with OptiMEM containing 5 μM calpeptin (Caymen Chemicals #14593), a pan calpain 1 and calpain 2 inhibitor, dissolved in DMSO or vehicle (DMSO) alone, at 37°C in 5% CO_2_. After 60 min, media containing calpeptin or vehicle was aspirated and replaced with OptiMEM containing 100 mM calcium chloride (CaCl_2_) to induce calpain activity or OptiMEM alone for a further 60 min.

For *CAPN1* knockdown experiments, media was aspirated from NSC-34 cells and replaced with a solution containing 10% OptiMEM, 90% DMEM with 10% FBS, 4 μL of Lipofectamine 2000 (Thermo Fisher Scientific), 2 μg of calpain sensor DNA and different concentrations of *CAPN1* siRNA or negative control siRNA (Origene, #SR319553). For *CAPN1* knockdown experiments, three different *CAPN1* siRNAs were combined in the provided buffer solution at a concentration of 1:10 and added to individual wells at a final concentration of 0 nM, 2 nM or 4 nM per well. For control siRNA experiments, the provided scrambled negative control siRNA was mixed in reaction buffer at a concentration of 1:10 and added to cells at a final concentration of 4 nM. NSC-34 cells were simultaneously transfected with calpain sensor or *CAPN1* siRNAs.

### Protein extraction and western blotting

At 24–26 h post-transient transfection, cell culture media was aspirated from NSC-34 cells and replaced with ice-cold radioimmunoprecipitation assay (RIPA) solution containing protease inhibitors (Complete Ultra tablets, Roche) and phosphatase inhibitors (PHOSstop tablets, Roche). Cells were incubated in RIPA solution for 15 min on ice before manual scraping was used to harvest cells. Cellular debris was removed from protein homogenates via centrifugation (15 min, 13 200g, 4°C). The cleared supernatant was collected, and protein concentration was determined using a Pierce BCA (bicinchonic acid) Protein Assay Kit (Thermo Fisher Scientific). Proteins were denatured by boiling at 95°C for 10 min.

Equal amounts of protein (12 μg) were prepared in Laemmli Buffer (BioRad) containing NuPAGE Reducing Agent (Life Technologies). Proteins were loaded and separated on NuPAGE 4–12% Bis-Tris gels (Thermo Fisher Scientific) via SDS-PAGE. Precision Plus Protein Dual Colour (Bio-Rad) was loaded as a molecular weight marker. Proteins were transferred to nitrocellulose membrane (#88018, Thermo Fisher Scientific) for immunoblot probing. Primary antibodies included: rabbit anti-GFP (1:2500, #ab290, Abcam) to identify full-length and cleaved calpain sensor protein bands due to sequence homology with CFP and YFP; rabbit anti-calpain 1 (1:2000, #2556, Cell Signalling Technology); and mouse anti-Beta Actin (1:5000, #A5441, Merck Life Science), as a protein loading control. Immunoblots were probed with LiCor fluorescent secondary antibodies (IRDye 800 goat anti-rabbit IgG or IRDye 680 goat anti-mouse 800, 1:10 000 dilution) and imaged using a LiCor Odyssey imaging system at the appropriate wavelengths. Band densitometry was quantified using Image Studio Lite, and the presence of calpain sensor or calpain 1 bands was normalized to beta actin expression or cleaved calpain sensor was normalized to full-length calpain sensor.

### Sensor cloning and vector production

The 1520 base pair coding sequence of the calpain sensor plasmid was excised using BsrGI (New England Biolabs #R3575S), Nhel (New England Biolabs #R3131S) restriction enzymes and rCutSmart^TM^ Buffer (New England Biolabs #B6004S) and subcloned into an AAV vector backbone (pAAV-EF1a-DIO-tdTomato-WPRE, a gift from Francois St-Pierre, Addgene plasmid # 202610; http://n2t.net/addgene:202610; RRID:Addgene_202610) after excision of the tdTomato sequence to produce pAAV-Ef1a-fDIO-calpain sensor-WPRE. After transformation, purification and sequence confirmation, pAAV-Ef1a-fDIO-calpain sensor-WPRE (titre 1.12 × 10^12^ vg/mL) and a control vector, pAAV-Ef1a-fDIO-tdTomato-WPRE (titre 2.33 × 10^12^ vg/mL), were packaged into AAV1 vectors using standard approaches (Vector and Genome Engineering Facility, Children’s Medical Research Institute, Sydney, Australia).

### Experimental animals

Animal experiments were approved by Macquarie University Animal Ethics Committee (Animal Research Authority: 2020/028). Male transgenic CMVMJD135 mice expressing human *ATXN3* (135 ± 2 CAG repeats, *n* = 6) and wildtype littermates (*n* = 9) were bred at Australian BioResources (Moss Vale, Australia), then transported to Macquarie University at 6–8 weeks of age. Mice were housed in individually ventilated cages (Techniplast) in temperature and humidity-controlled rooms on a 12-h light–dark cycle (lights off at 6pm). Animals were provided environmental enrichment and *ad libitum* access to standard chow and water. Mice were given a minimum of 5 days to acclimate to Macquarie University before commencing experimentation.

### Stereotaxic surgery

Mice were anaesthetized with isoflurane in medical oxygen (5% induction, 1–3% maintenance, 1 L/min flow rate) and placed into a stereotaxic frame. Body temperature was maintained at 37°C using a thermostatically controlled heating pad. Saline (0.3 mL, s.c.), analgesic (carprofen, 5 mg/kg, s.c.), and prophylactic antibiotics (cephazolin 20 mg/kg, i.m.) were administered. Depth of anaesthesia was monitored every 15 min via assessment of respiration rate and hind paw reflex.

The scalp was shaved and cleaned with chlorohexidine solution. The skull surface was exposed via midline incision, cleaned with hydrogen peroxide solution, and scored using a scalpel blade to enhance adhesion. A small hole was bored overlying the cerebellum for viral injection and fibre implantation.

Three vectors were mixed and co-injected in each animal: AAV1-Ef1a-DIO-calpain sensor to measure FRET signals as a proxy for calpain activity; AAV1-Ef1a-DIO-tdTomato to mark vector injection sites; and AAV1-hSyn1-cre (Addgene 105553, 7 × 10^12^ vg/mL) to drive cre expression exclusively within neurons. Vectors were gently mixed in a ratio of 2:1:1, respectively, and injected into the midline cerebellum 6.25 mm caudal to Bregma. A small injection (∼100 nL) was performed 2.1 mm below the skull surface. The glass pipette was held in place for ∼5 min and raised to a position of 2.0 mm below the skull surface, where 250 nL was injected at a rate of 50 nL per minute. The micropipette was held in place for 10 min after injection and then withdrawn. Next, a fibre optic cannula (1.25 mm ferrule diameter, 200 μm fibre core, numerical aperture 0.37, Neurophotometrics) was inserted 2.0 mm below the skull surface. Cyanoacrylate glue and dental cement were used to secure the fibre optrode in place.

### Postoperative monitoring

Mice were recovered from isoflurane anaesthetic and closely monitored for 3–5 days. During their recovery, mice received antibiotics (cephazolin 20 mg/kg, i.m.) prophylactically for 2 days, carprofen (5 mg/kg, s.c.) for pain relief, saline (0.3 mL, s.c.) for dehydration and Sustagen (0.3 mL, p.o.) for constipation, when required. Mice that did not return to pre-surgery body weight within 7 days were euthanized due to poor postoperative recovery. Throughout the experiment, we observed a higher-than-expected mortality rate, with some transgenic CMVMJD135 animals failing to survive the initial postoperative period, requiring euthanasia.

### Fibre photometry experimental set-up

A custom-built fibre photometry apparatus was mounted on an optical bench (Thorlabs, MB1824). The apparatus consisted of multiple dichroic cube holders (Thorlabs, DFM1) equipped with lenses (30 mm lens, Thorlabs, AC254-030-A-ML), emission filters (Thorlabs, FB410-10), dichroic mirrors (FF20-Di02-25x36) or an LED (415 nm, Thorlabs, M415L4). The 415 nm LED was used to excite ECFP (excitation = 434 nm, emission = 477 nm), which would transfer electrons (via FRET) to EYFP (excitation = 513 nm, emission = 527 nm), producing EYFP emission. A multi-band dichroic beamsplitter (Semrock, FF410/504/582/669-Di01-25x36) was used to direct the LED wavelengths into a 20x/0.5-NA Plan Fluorite objective (Thorlabs, RMS20X-PF). The patch cable (1.25 mm/200 μm, Neurophotometrics) was coupled to a 10 mm mono-fibre optic cannula implanted within the mouse brain via ceramic mating sleeves (Thorlabs, ADAL-5). A dichroic beam splitter (Semrock, FF555-Di03-25x36) directed < 550 nM light emitted from the brain through a bandpass filter (FF01-520/35-25, Semrock) and through a light-impenetrable tube onto a monochrome sCMOS camera (FLIR, BLFY-U3-23S6M-C; 81% quantum efficiency), which recorded ∼41 frames per second. Therefore, the camera was sensitive only to light emitted from the brain between 502.5 and 537.5 nM (FRET EFYP emission wavelength is centred on 527 nM). The entire fibre photometry apparatus was covered with black rubberized fabric (Doric, BK5) to ensure no ambient light affected photometry recordings. The 415 nm LED was used at 50% power (0.7 μw).

Using an open-source template (https://github.com/neurophotometrics), a custom modified Bonsai program was created to record fluorescent intensity from a rectangular region of interest within the fibre face projected onto the camera sensor. At the end of each recording session, Bonsai produced an .xlsx file where each row represented a frame from the camera and a column containing pixel intensity values (intensity of fluorescent light between 502.5 and 537.5 nm). Camera frames were captured in synchrony with excitation of the 415 nM LED, interleaved with frames in which the LED was off, serving as a control for fluctuating light due to motion within the recording period.

### Drug treatments

**Figure 2:**
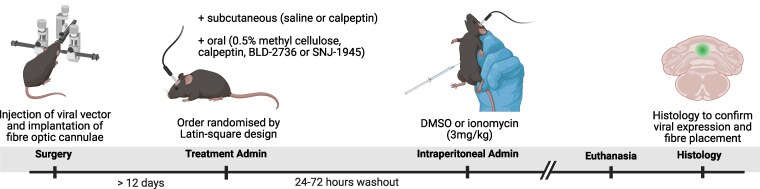
**Timeline for animal experiments, including treatment administration.** This figure was created in BioRender. Available at https://BioRender.com/36j9p7l.

Drug treatments were administered to the mice as indicated in Fig. 2. Calpeptin (Caymen Chemicals #14593), a pan calpain 1 and calpain 2 inhibitor, was dissolved in either 0.9% saline or 0.5% methyl cellulose (Sigma #M0387) at 2 mg/mL and stored at −20°C until use. Animals were administered doses of calpeptin ranging between 0.2 and 2 mg/kg, doses previously reported to inhibit spectrin cleavage^75^ and decrease expression of calpain 1 and calpain 2.^76^ BLD-2736, an inhibitor of calpains 1, 2 and 9 synthesized by Blade Therapeutics, was dissolved in 0.5% methyl cellulose and administered orally at doses ranging from 40 to 100 mg/kg. SNJ-1945, a pan calpain inhibitor (MedKoo Biosciences Inc #564979), was dissolved in 0.5% methyl cellulose at 50 mg/mL and stored at 4°C. SNJ-1945 was administered at a dose of 50 mg/kg, a dose that has previously been reported to inhibit calpains.^77^ Ionomycin (Sapphire Bioscience #190-04974), an antibiotic known to alter calcium signalling was dissolved in DMSO at 3 mg/mL and stored at −20°C. Ionomycin was administered at a dose of 3 mg/kg via intraperitoneal injection. Administration of vehicle alone was used as a control. Drug treatments were administered once daily at a volume of 10 mL/kg according to body weight. The order of administered compounds and doses was determined using a Latin-square design to prevent ordering effects. An exception to this was ionomycin treatments, which were administered after all other testing was complete, to prevent premature quenching of the calpain sensor construct. Experimenters were not blinded to drug conditions.

### Experimental protocol

Before commencing fibre photometry recordings, mice were weighed, and their body weight was recorded. Next, implanted fibre optic cannula was gently cleaned with 80% ethanol before attaching to ceramic mating sleeves coupled to the patch cord. Mice were attached to the patch cord and briefly habituated. Recording involved 1 min with no LED excitation (off signal) followed by 1 min of interleaved 415 LED excitation and control (no LED excitation). After conducting a pre-treatment recording, mice were immediately administered compounds. Post-treatment recordings were performed at 1-, 2- and 3-h post-administration, following the same procedure as the pre-treatment recording. Animals were given a period of 24–72 h for treatment washout between recording sessions.

### Analysis of fibre photometry data

To identify any confounds of animal movement or other excitation-independent artefacts in the detected signal, we alternated between pixel intensity during 415 nm excitation and no excitation (off signal), which was used as a control. FRET was calculated by averaging the captured EYFP (500–540 nm) signal intensity measurements over a 60 s period of 415 nm LED excitation (∼2000 unique fluorescent intensity measurements) and subtracting the interleaved control signal. To examine the effect of calpain inhibitor treatments, a pre-treatment FRET signal was calculated at time 0, prior to drug administration. All subsequent measurements were expressed as ΔFRET relative to baseline FRET [(FRET – pre-treatment FRET)/pre-treatment FRET) × 100]. This calculation of ΔFRET is identical to that performed by Jones-Tabah *et al*.^78^ The maximum ΔFRET represents the highest ΔFRET value observed during the recording day (up to 3 h post-administration). As ionomycin was expected to activate calpains and thus decrease FRET signal, we also calculated the minimum ΔFRET for this treatment. Area under the curve was calculated using the trapezoidal rule, following the equation [(ΔFRET timepoint_A_ + ΔFRET timepoint_B_)/2 × (timepoint_B_ – timepoint_A_)] for the data for each hour within the recording, followed by summing the resulting values from the 3 h.

### Euthanasia and histology

At the conclusion of experiments, mice were euthanized via injection of sodium pentobarbitone (300 mg/kg, i.p.) and were transcardially perfused with 0.9% saline and 4% paraformaldehyde diluted in PBS once reflexes were absent. Brains were extracted and post-fixed at 4°C for a further 24 h, then transferred to 30% sucrose diluted in PBS for a minimum of 24 h before cryopreservation.

To examine viral expression, brains were sectioned coronally (50 μm thick, 1:4 series) using a vibrating microtome (Leica Microsystems, VTS1200S). Sections were mounted and coverslipped with Dako fluorescence mounting media (Agilent #S3023) and imaged using an Olympus VS200 Slide Scanner (10× objective). To visualize AAV-calpain sensor expression, brain sections were imaged under 488/FITC fluorescence. The number of neurons expressing AAV1-calpain sensor was manually counted using Image J. Animals with <100 EYFP^+^ neurons (*n* = 1), or in which there was off-target fibre placement, were excluded (*n* = 3).

### Statistical analysis

Statistical analysis was performed using GraphPad Prism (v 10.1.2). One-way ANOVAs were used to determine the effects of genetic manipulation of *CAPN1* (overexpression or knockdown with *CAPN1* siRNA) or calpeptin treatment in calpain-sensor expressing NSC-34 cells with *post hoc* multiple comparisons. A two-way ANOVA (factor 1: transfection with calpain sensor, factor 2: transfection with p3xFlag-*CAPN1*) with Tukey’s *post hoc* comparisons was used to determine the effects of calpain 1-mediated cleavage of the calpain sensor in NSC-34 cells. To determine if administered compounds affected FRET signal in fibre photometry experiments, ΔFRET values were analysed using a two-way repeated measures ANOVA (factor 1: time; factor 2: treatment). In cases where values were missing, a mixed effects two-way ANOVA was used. One-way ANOVA with Tukey’s *post hoc* tests was used to compare differences in the maximum ΔFRET across different doses of the same treatment. When only one treatment dose was compared with control, a paired or an unpaired *t*-test was used. Matched experimental data were analysed, i.e. only experimental animals that were exposed to all treatment doses were included in the analysis for that compound. Pearson’s correlations were used to examine the relationship between the number of virally transduced neurons at euthanasia and the number of days between viral transduction and euthanasia or the number of fibre photometry test sessions. Statistical significance is defined as *P* < 0.05. As fewer transgenic CMVMJD135 animals modelling SCA3 survived the surgical procedure than initially expected, our SCA3 animal sample size (*n* = 6) was underpowered compared with wildtype animals (*n* = 9), limiting our ability to analyse genotype effects. Data from wildtype animals were combined with data from SCA3 animals, and statistical analysis was performed on combined datasets to maintain statistical power.

## Results

### *In silico* prediction of calpain sensor linking sequence cleavage

We utilized a newly developed tool, ProsperousPlus,^73^ to explore whether proteases other than calpains may be capable of cleaving the amino acid linking sequence separating the ECFP and EYFP fluorophores within the calpain sensor. ProsperousPlus, a freely available online platform (http://prosperousplus.unimelb-biotools.cloud.edu.au/index.php/prediction), provided cleavage prediction scores for calpain 1 and 2, several caspase isoforms and granzyme B. A prediction score close to 1 is indicative of high certainty that the selected protease will cleave the amino acid sequence, whilst a score of 0 indicates no cleavage. ProsperousPlus provided prediction scores of 0.59 for calpain 1 and 0.58 for calpain 2, confirming the validity of the calpain sensor construct. As the linking sequence was derived from α-spectrin, a protein that can be cleaved by caspase proteases, we first evaluated the prediction score for caspases 1–3, 6–8. The prediction score for caspase-1 was 0.486; however, all other caspases obtained a prediction score of below 0.25, confirming that caspases were unlikely to cleave the calpain sensor. Lastly, we investigated whether granzyme-B was likely to cleave the calpain sensor linking sequence. ProsperousPlus provided a prediction score of 0.326. Collectively, these findings suggest that calpains are mostly likely of the three protease families examined to cleave the linking sequence; however, the linking sequence may still be cleaved by other proteases families not examined within this analysis.

### Modulation of calpain activity *in vitro* affected calpain sensor cleavage

**Figure 3:**
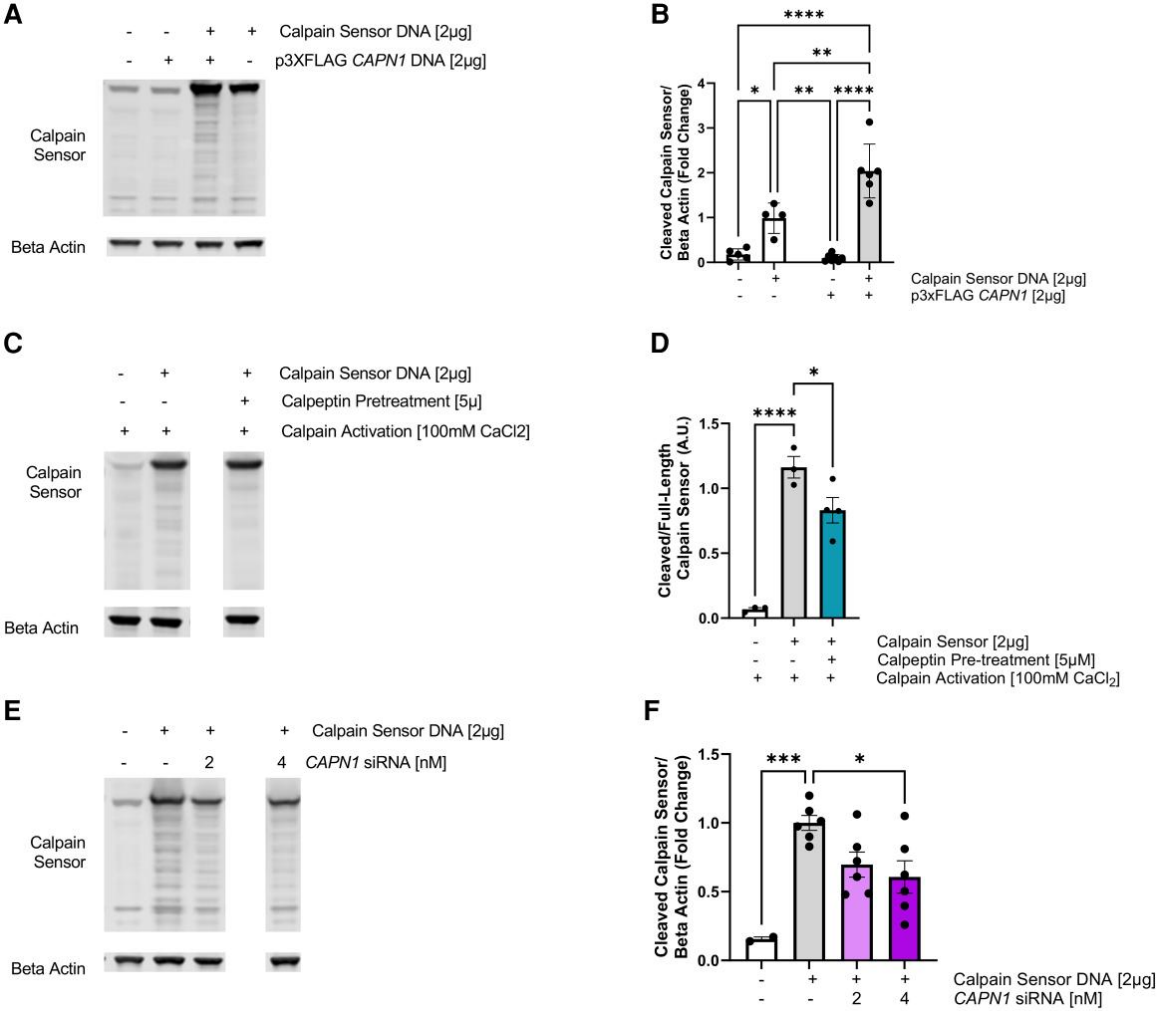
**Validation of calpain sensor construct in cultured neuronal-like NSC-34 cells.** (**A**) Representative western blot image of full-length and cleaved calpain sensor in NSC-34 cells expressing calpain sensor and p3xFLAG *CAPN1*. (**B**) Analysis of the amount of cleaved calpain sensor relative to loading control (beta actin). (**C**) Representative western blot of calpain sensor expression and cleavage across untransfected NSC-34 cells and NSC-34 cells pre-treated with 5 μM calpeptin or vehicle prior to calcium chloride (100 mM) exposure. (**D**) Quantitative analysis of the amount of cleaved calpain sensor relative to full-length calpain sensor in cells exposed to calcium chloride together with/without calpeptin treatment. (**E**) Representative western blot of calpain sensor abundance in untransfected NSC-34 cells, NSC-34 cells expressing calpain sensor and NSC-34 cells expressing calpain sensor and *CAPN1* siRNA (2 or 4 nM). (**F**) Quantitative analysis of the amount of cleaved calpain sensor in cells expressing calpain sensor construct and *CAPN1* siRNA. Data were analysed using either one-way or two-way ANOVAs with *post hoc* multiple comparisons. (**B–F**) Display the group mean and SEM. * represents *P* < 0.05 and ** represents *P* < 0.01, *** represents *P* < 0.001 and **** represents *P* < 0.0001. Each dot point represents immunoblot band density from an independent experiment (*n* = 3–6 per group). See Supplementary material for uncropped western blot images.

To validate the calpain sensor previously developed by Stockholm *et al.*^66^ and confirm whether genetic manipulation of calpain 1 or treatment with calpeptin could modulate calpain sensor cleavage, we transiently transfected cultured NSC-34 (neuronal-like) cells with the calpain sensor construct and genetically modified levels of calpain 1, via calpain 1 overexpression or knockdown using *CAPN1* siRNA, and examined the effect of calpeptin pre-treatment (5 μM) and exposure to CaCl_2_ (100 mM) on calpain sensor cleavage. First, we transfected cultured NSC-34 cells with p3xFLAG-*CAPN1* to overexpress human calpain 1 protein and calpain sensor. Immunoblotting of NSC-34 protein lysates expressing p3XFLAG *CAPN1* and/or calpain sensor and probing for GFP revealed the presence of a strong band at the expected height of full-length calpain sensor (∼45 kDa) and several cleavage fragments at lower molecular weight (Fig. 3A). Quantification and statistical analysis of these cleavage fragments via two-way ANOVA revealed a statistically significant effect of calpain sensor expression (*F*(1, 18) = 79.72, *P* < 0.0001), a statistically significant effect of *CAPN1* overexpression (*F*(1, 18) = 9.989, *P* = 0.0054) and a statistically significant interaction effect (*F*(1, 18) = 13.51, *P* = 0.0017), whereby co-expression of calpain sensor and *CAPN1* increased the presence of cleaved calpain sensor relative to beta actin (Fig. 3B). To confirm that the p3xFLAG-*CAPN1* construct was indeed leading to overexpression of calpain 1 protein as expected, we transfected NSC-34 cells with different concentrations of p3xFLAG-*CAPN1* DNA and extracted protein. Immunoblotting of NSC-34 cells overexpressing *CAPN1* revealed differential expression of calpain 1 bands (∼80–76 kDa) relative to the loading control beta actin across transfection conditions (Supplementary Fig. 1A). One-way ANOVA revealed a statistically significant difference across groups (*F*(2, 8) = 34.37, *P* = 0.0001, Supplementary Fig. 1B). *Post hoc* multiple comparisons revealed increased levels of calpain 1 protein following transfection with 1 and 2 µg p3xFLAG-*CAPN1* when compared to untransfected controls (*P* = 0.0128 and *P* = 0.0001, respectively). Further, calpain 1 protein levels were increased in NSC-34 cells transfected with 2 µg p3xFLAG-*CAPN1* when compared to 1 µg DNA (*P* = 0.0026). When considered together, these findings suggest that calpain 1 overexpression leads to enhanced calpain sensor cleavage when compared to cells expressing endogenous calpains only.

Next, we explored whether calpeptin treatment could reduce calpain sensor cleavage. Immunoblotting of protein lysates from NSC-34 cells exposed to calcium chloride for calpain activation revealed the presence of full-length calpain sensor protein and GFP-positive cleavage fragments in vehicle and calpeptin-treated cells (Fig. 3C), similar to what was observed following overexpression of calpain 1. Quantification and one-way ANOVA analysis of cleaved calpain sensor relative to full-length revealed a statistically significant difference across groups (*F*(2, 7) = 42.27, *P* = 0.0001). *Post hoc* multiple comparisons revealed increased presence of cleaved calpain sensor in vehicle-treated NSC-34 cells compared with untransfected control cells and calpeptin-treated cells (*P* < 0.0001 and *P* = 0.0406, respectively, Fig. 3D).

Lastly, to confirm whether the observed calpain sensor cleavage effects were due to calpain 1 activity, we used a *CAPN1* siRNA to knockdown calpain 1 expression in NSC-34 cells. Immunoblotting of NSC-34 cell lysates treated with *CAPN1* siRNA revealed decreased presence of cleaved calpain sensor fragments (Fig. 3E). Densiometric quantification followed by one-way ANOVA revealed a statistically significant difference in calpain sensor cleavage across treatment groups, (*F*(5, 22) = 5.680, *P* = 0.0016, Fig. 3F). *Post hoc* multiple comparisons revealed a statistically significant increase in calpain sensor cleavage in negative control siRNA-treated cells compared when to untransfected controls (*P* = 0.0003). Further, decreased calpain sensor cleavage was observed when negative control siRNA-treated cells were compared to NSC-34 cells treated with 4 nM *CAPN1* siRNA (*P* = 0.0150). The comparison between negative control siRNA-treated cells and cells treated with 2 nM *CAPN1* siRNA did not reach statistical significance (*P* = 0.0773). To confirm the CAPN1 siRNA was working as expected to knockdown levels of calpain 1 protein, we also performed immunoblotting of siRNA-treated NSC-34 lysates and probed for calpain 1. As expected, immunoblotting of these lysates revealed a reduction in calpain 1 levels following exposure to *CAPN1* siRNA (Supplementary Fig. 1C), confirming the efficacy of the CAPN1 siRNA. Densiometric analysis of calpain 1 protein levels across groups revealed a statistically significant difference (*F*(5, 12) = 5.024, *P* = 0.0103, Supplementary Fig. 1D). *Post hoc* multiple comparisons confirmed a statistically significant reduction in calpain 1 protein levels following treatment with 2 and 4 nM *CAPN1* siRNA when compared to cells treated with a negative control scramble siRNA (*P* = 0.0045 and *P* = 0.0249, respectively).

Collectively, these findings provide *in vitro* validation of the utility of the calpain sensor construct as a tool to investigate modulators of calpain activity, as cleavage of the calpain sensor construct is amenable to *CAPN1* genetic manipulation and treatment with small molecules known to modulate the activity of calpain 1.

### Subcutaneous administration of calpeptin increased FRET signal

**Figure 4:**
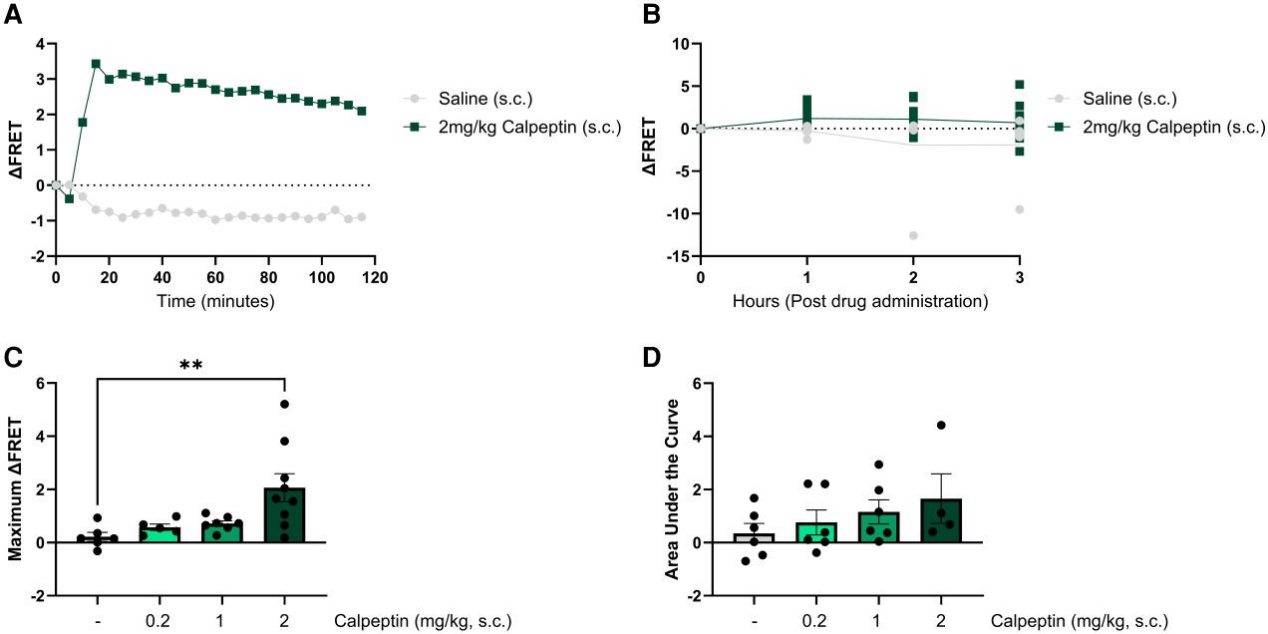
**Subcutaneous administration of calpeptin changes FRET signal within the cerebellum.** (**A**) Matched traces from one representative experimental animal in response to vehicle or calpeptin treatment over a 2-h period. (**B**) Change in FRET signal over a 3-h period following subcutaneous (s.c.) administration of vehicle or calpeptin. (**C**) The maximum ΔFRET value detected within a 3-h recording window following subcutaneous administration of vehicle or calpeptin. (**D**) The calculated area under the curve in the 3 h immediately following subcutaneous administration of vehicle or calpeptin. (**B**)–(**D**) Display the group mean and SEM. ** represents *P* < 0.01. Data were statistically analysed using a two-way ANOVA (**B**) or one-way ANOVA (**C** and **D**). Each dot point represents the FRET signal detected in a single experimental animal (*n* = 4–7 per treatment group).

Following our *in vitro* validation, we next aimed to investigate the effects of calpeptin administration on calpain sensor expression *in vivo*. We first examined the effect of subcutaneously administered calpeptin (2 mg/kg) versus saline control, using a continuous recording for 120 min. Following administration of calpeptin, we detected a distinct elevation in FRET signal compared to pre-treatment levels (Fig. 4A). The change in FRET signal was apparent within 10 min of calpeptin administration and remained elevated for the entire 120-min recording period. One week later, we administered 0.9% saline (s.c.) to the same animal and detected a small negative ΔFRET, which remained stable from 20 to 120 min post-administration, suggesting that the change in calpain activity was relatively constant once calpeptin had taken effect. However, continuous 120-min recordings proved technically challenging due to patch cord detachment.

As the change in FRET signal appeared relatively stable from 20 to 120 min post-administration, and to allow greater testing capacity (i.e. more experimental animals) over a longer testing period, recording parameters were changed to instead record for 2 min each hour from each experimental animal. Similar to continuous recordings, we detected a positive ΔFRET following treatment with 2 mg/kg calpeptin (s.c.) and ΔFRET signal that neared 0 (no change from pre-administration) or became slightly negative in response to saline vehicle. Two-way RM ANOVA revealed a statistically significant effect of treatment (*F*(1, 14) = 4.675, *P* = 0.0484, Fig. 4B). Analysis of the effect of time or treatment × time interaction effect did not reveal any statistically significant differences (*F*(1, 14) = 1.046, *P* = 0.3417 and *F*(3, 40) = 1.886, *P* = 0.1475, respectively). Comparison of the maximum ΔFRET following administration of three doses of calpeptin (0.2, 1 or 2 mg/kg, s.c.) compared to vehicle (0.9% saline, s.c.) revealed a statistically significant difference across the doses (*F*(3, 23) = 5.442, *P* = 0.0056). *Post hoc* analysis revealed an effect of dose, whereby treatment with 2 mg/kg calpeptin (s.c.) produced a statistically significant increase in the maximum ΔFRET when compared to vehicle treatment (*P* = 0.00396), whilst treatment with lower doses, 0.2 mg/kg or 1 mg/kg calpeptin (s.c.), did not significantly differ from vehicle treatment (*P* = 0.8651 and *P* = 0.6728, respectively, Fig. 4C). We observed a dose-dependent increase in the area under the curve for all three doses of calpeptin, however no statistical significance was detected (*F*(3, 18) = 0.1384, *P* = 0.4144, Fig. 4D).

### Oral administration of calpeptin, BLD-2736 and SNJ-1945 increased FRET signals

**Figure 5:**
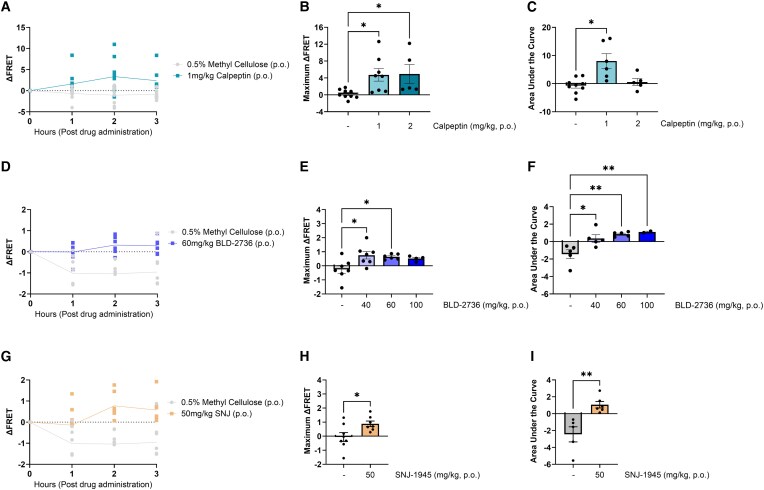
**Orally administered calpain inhibitor compounds increased cerebellar FRET signalling.** (**A**) 1 mg/kg calpeptin administered orally (p.o.) increased ΔFRET compared to vehicle. (**B**) Maximum ΔFRET was significantly increased following 1 and 2 mg/kg calpeptin. (**C**) Area under the curve was significantly increased for 1 mg/kg calpeptin compared to vehicle, but not 2 mg/kg calpeptin. (**D**) 60 mg/kg BLD-2736 increased ΔFRET compared to vehicle. (**E**) Maximum ΔFRET was significantly increased following 40 and 60 mg/kg BLD-2736 compared to vehicle. (**F**) Area under the curve was significantly increased for all doses of BLD-2736 compared to vehicle. (**G**) 50 mg/kg SNJ-1945 increased ΔFRET compared to vehicle. (**H**) Maximum ΔFRET was significantly increased following 50 mg/kg SNJ-1945 (paired *t*-test) compared to vehicle. (**I**) Area under the curve was significantly increased for 50 mg/kg SNJ-1945 compared to vehicle. All graphs display the group mean and SEM. * represents *P* < 0.05 and ** represents *P* < 0.01. Data were statistically analysed using a two-way ANOVA (**A**, **D**, **G**), one-way ANOVA (**B**, **C**, **E**, **F**) or paired *t*-test (**H**, **I**). Each dot point represents the FRET signal detected in a single experimental animal (*n* = 4–9 per treatment group).

We also tested whether oral administration of calpain inhibitors could alter cerebellar FRET signals. Oral administration of 1 mg/kg calpeptin produced a significant elevation in FRET signal compared to 0.5% methyl cellulose vehicle, with two-way RM ANOVA revealing a statistically significant effect of treatment (*F*(1, 18) = 13.02, *P* = 0.0020) and a time × treatment interaction effect (*F*(3, 50) = 5.884, *P* = 0.0016, Fig. 5A). The effect of time was not found to be statistically significant (*F*(1, 18) = 1.586, *P* = 0.2185). Interestingly, administration of 2 mg/kg calpeptin produces a completely different response to 1 mg/kg, producing a positive ΔFRET at 1-h post-administration, however at 2 h post-administration ΔFRET returns to pre-treatment levels and changes to become more negative at 3 h post-administration (Supplementary Fig. 2). One-way ANOVA of the maximum ΔFRET values revealed a significant difference across treatment groups (*F*(2, 19) = 2.012, *P* = 0.0108). *Post hoc* comparisons revealed a statistically significant increase in the maximum ΔFRET following treatment with 1 mg/kg and 2 mg/kg calpeptin compared to vehicle (0.5% methyl cellulose) treatment (*P* = 0.0286 and *P* = 0.0273, respectively; Fig. 5B). Analysis of the area under the curve revealed a statistically significant difference across treatment groups (*P* = 0.0115). *Post hoc* comparisons revealed that treatment with 1 mg/kg calpeptin produced an increased area under the curve compared to vehicle treatment (*P* = 0.0028); however, treatment with 2 mg/kg calpeptin did not significantly differ from vehicle treatment (*P* = 0.8808, Fig. 5C).

To explore the relative effectiveness of different calpain inhibitor compounds in our assay, we used a similar approach to examine the effects of two other calpain inhibitors, BLD-2736 and SNJ-1945, on FRET signal. Oral administration of BLD-2736 produced a positive increase in FRET signal, with two-way RM ANOVA revealing a statistically significant effect of treatment (*F*(1, 11) = 13.99, *P* = 0.0033) and time × treatment interaction (*F*(3, 33) = 3.974, *P* = 0.0160, Fig. 5D). We did not detect any significant effect of time (*F*(1, 11) = 1.941, *P* = 0.1456). Analysis of the maximum ΔFRET revealed an effect of BLD-2736 treatment (*F*(3, 20) = 4.336, *P* = 0.0165). *Post hoc* comparisons revealed a statistically significant increase in ΔFRET following administration of 40 mg/kg and 60 mg/kg BLD-2736 doses (*P* = 0.0102 and *P* = 0.0290, respectively) but not 100 mg/kg (*P* = 0.1110) when compared to vehicle treatment (Fig. 5E). Analysis of area under the curve revealed a significant increase in FRET values following BLD-2736 treatment (*F*(3, 13) = 8.105, *P* = 0.0027). *Post hoc* comparisons revealed a statistically significant increase in the area under the curve with all three doses of BLD-2736 when compared to vehicle treatment (versus 40 mg/kg BLD-2736, *P* = 0.0127, versus 60 mg/kg BLD-2736, *P* = 0.0022, versus 100 mg/kg BLD-2736, *P* = 0.0085, Fig. 5F).

Oral administration of 50 mg/kg SNJ-1945 produced a positive effect on FRET signal, with two-way RM ANOVA revealing a statistically significant effect of treatment (*F*(1, 12) = 12.72, *P* = 0.0285) and a treatment × time interaction effect (*F*(3, 36) = 5.345, *P* = 0.0182, Fig. 5G). We did not detect any significant effect of time (*F*(1, 12) = 2.038, *P* = 0.2335). Analysis comparing the maximum ΔFRET produced by SNJ-1945 and vehicle revealed a statistically significant difference, with SNJ-1945 producing an increased maximum ΔFRET value (*t*(13) = 2.459, *P* = 0.0287, Fig. 5H). Analysis of the area under the curve revealed a statistically significant increase with SNJ-1945 treatment when compared to vehicle treatment (*t*(9) = 3.837, *P* = 0.0040, Fig. 5I).

### Intraperitoneal administration of ionomycin reduced FRET signal

**Figure 6:**
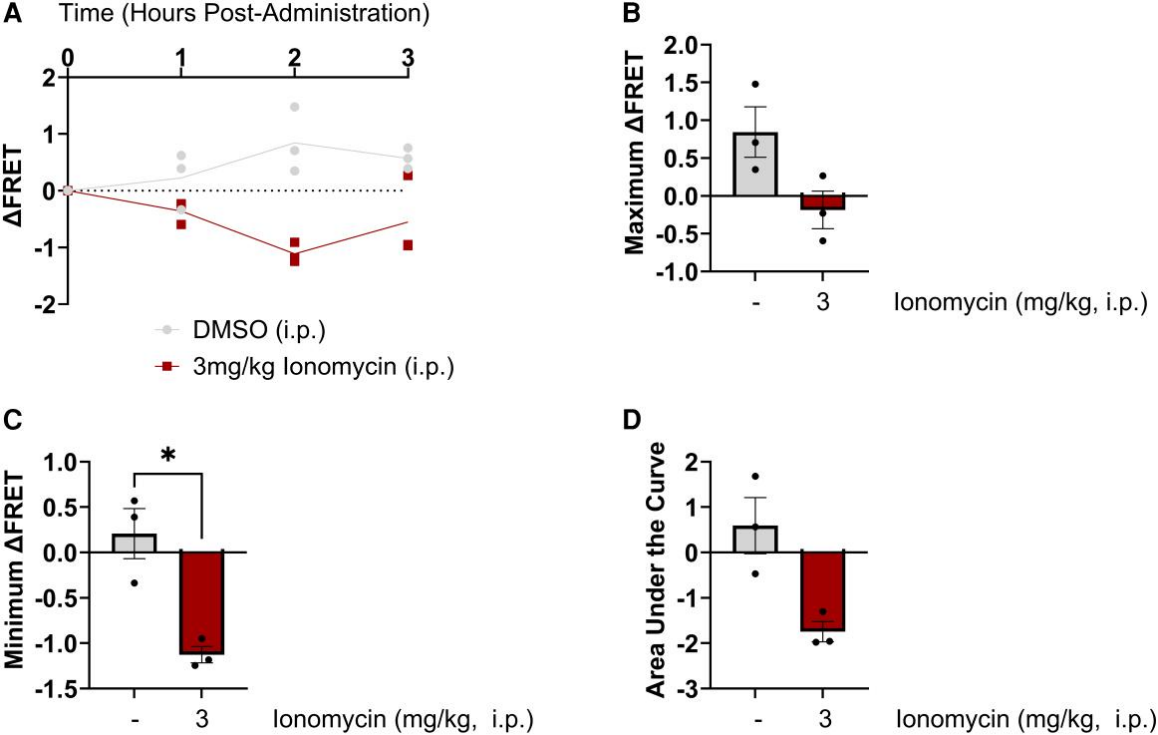
**Treatment with ionomycin, an antibiotic known to increase calcium concentrations, was found to decrease the detected Förster resonance energy transfer (FRET) signal, indicating increased calpain activity.** (**A**) Intraperitoneal (i.p.) administration of 3 mg/kg ionomycin was found to decrease FRET signal when compared to vehicle treatment. (**B**) Analysis of the maximum change in FRET (ΔFRET) signal revealed no statistically significant differences between ionomycin and vehicle treatments. (**C**) Analysis of the minimum ΔFRET revealed a statistically significant decrease with ionomycin treatment. (**D**) Area under the curve analysis also revealed a decrease in FRET signal with ionomycin treatment; however, this failed to reach statistical significance. All graphs display the group mean and SEM. * represents *P* < 0.05, # represents *P* = 0.0787. Data were statistically analysed using a two-way ANOVA (**A**) or paired *t*-tests (**B–D**). Each dot point represents the FRET signal detected in a single experimental animal (*n* = 3 per treatment group).

Next, we wanted to examine whether administration of a compound that increases intracellular calcium concentrations would affect FRET signals. We chose ionomycin, a calcium ionophore,^79^ for this purpose. Treatment with 3 mg/kg ionomycin produced a decrease in FRET signal when compared to vehicle (DMSO) treatment, with two-way RM ANOVA revealing a statistically significant effect of treatment (*F*(1, 4) = 21.98, *P* = 0.0094) and a significant time × treatment interaction effect (*F*(3, 12) = 8.468, *P* = 0.0027, Fig. 6A). We did not detect any significant effect of time overall (*F*(1, 4) = 0.2193, *P* = 0.8466). A paired *t*-test revealed a non-significant difference between the maximum ΔFRET produced by vehicle and ionomycin treatments (*t*(2) = 1.784, *P* = 0.2164, Fig. 6B). As ionomycin was predicted to decrease FRET signal, we also examined the minimum ΔFRET value. Analysis of the minimum ΔFRET value produced following vehicle or ionomycin treatment revealed a statistically significant decrease with ionomycin treatment (*t*(2) = 4.771, *P* = 0.0427, Fig. 6C), although area under the curve measurements were not statistically different (*t*(2) = 2.848, *P* = 0.1044, Fig. 6D). We observed that injection of DMSO produced a slightly higher ΔFRET compared to other vehicles (0.5% methyl cellulose or saline) however two-way RM ANOVA revealed a non-significant effect of vehicle (*F*(3, 13) = 1.506, *P* = 0.2595, data not shown). Analysis of the maximum ΔFRET produced by each vehicle via one-way ANOVA also revealed a lack of statistical significance between groups (*F*(3, 19) = 0.9136, *P* = 0.4530, data not shown).

### Histological analysis of viral expression

**Figure 7:**
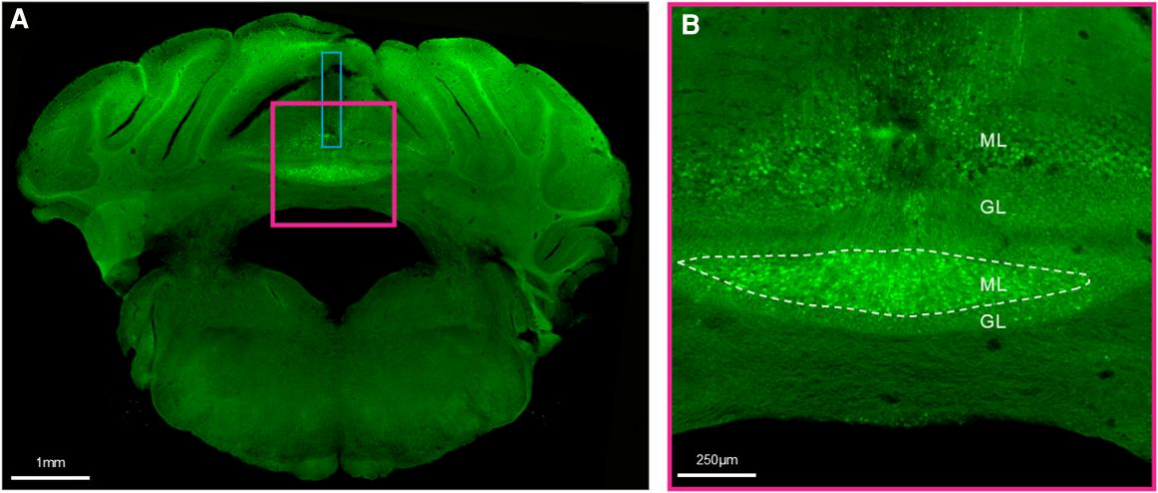
**Representative images showing viral expression of calpain sensor in molecular and granule cell layers of the cerebellum.** (**A**) Low magnification (10× objective) image depicting AAV1-calpain sensor (green) expression within the cerebellum. Blue box depicts the location of the implanted fibre optic cannula. (**B**) Higher magnification image of the region within the pink square in (**A**). Abbreviations: ML, molecular layer; GL, granule cell layer.

Histological analysis of sensor expression revealed that most viral expression was within the anterior lobules (lobules III and IV/V) of the cerebellum (Fig. 7). We observed the highest density of calpain-sensor expressing neurons within the molecular layer, with some sparse expression in the Purkinje cell layer and granular layer. We also observed some sparse expression within deep cerebellar nuclei and diffuse expression in cerebellar fibre tracts. To confirm whether there was any relationship between the amount of calpain sensor virus present at euthanasia and the time between viral transduction and euthanasia, we performed a Pearson’s correlation analysis. Viral expression at euthanasia was found to be correlated with the length of time between viral transduction surgery and euthanasia sessions (*R*^2^ = 0.2513, *P* = 0.0404, Supplementary Fig. 3), suggesting calpain sensor expression may decrease with time.

## Discussion

### Summary of main findings

In this study, we first confirmed the validity and specificity of the calpain sensor, using the *in silico* tool ProsperousPlus to assess the likelihood of protease cleavage, comparing prediction scores of calpain, caspase and granzyme B cleavage of the amino acid sequence linking ECFP and EFYP. Of these protease families, calpains were found to have the highest prediction score, with caspase isoforms and granzyme B less likely to cleave the calpain sensor, confirming specificity and validity of the sensor. To validate the calpain sensor construct *in vitro*, we examined whether genetic manipulation of *CAPN1* levels or calpeptin pre-treatment could alter calpain-mediated cleavage of the calpain sensor construct in cultured neuronal-like NSC-34 cells. Our cell culture validation experiments confirmed that (i) overexpression of calpain 1 increased the presence of calpain 1 protein and calpain sensor cleavage; (ii) pre-treatment with calpeptin reduced cleavage of the calpain sensor construct in NSC-34 cells; and (iii) genetic knockdown of calpain 1 using a *CAPN1* siRNA decreased the presence of calpain 1 protein and cleaved calpain sensor. These *in vitro* findings confirmed the suitability of the calpain sensor construct to examine the effects of small molecules expected to modulate calpain activity within neuronal-like cells.

Next, we developed a novel methodology that could be used to explore neuronal calpain activity and test the effect of calpain inhibitor compounds *in vivo*. This novel methodology was used to detect time-dependent changes in neuronal calpain activity across repeated test sessions within the same experimental animal. To our knowledge, this is the first report of a methodology that enables repeated examination of neuronal calpain activity *in vivo*, thereby enabling direct comparison of the pharmacokinetics of calpain inhibitors with their pharmacodynamics, facilitating novel drug development in this field. We chose calpeptin as a compound to initially test the capability of our novel methodology, as this compound has been proven to decrease calpain activity in models of spinocerebellar ataxia-3,^48,68,69^ spinal muscular atrophy,^80,81^ multiple sclerosis^82^ and Parkinson’s disease.^40^

From continuous recordings, we found that subcutaneously administered calpeptin (2 mg/kg) produced an increase in ΔFRET from 5 min post-administration, which remained stable from 20 to 120 min post-administration. However, continuous recording proved challenging with movement from freely behaving animals, leading to patch cord detachment. Considering we saw a rapid increase in ΔFRET from 5 to 15 min post-administration, then a stabilization in FRET signal from 20 to 120 min post-administration, we instead recorded for shorter periods (maximum of 2 min) every hour for a total of 3 h following drug administration. This minimized challenges associated with patch cord detachment, limiting the potential confounding impacts of photobleaching on signal decay by reducing the total excitation time, and reduced the amount of high-level supervision imposed by continuous recording sessions, enabling more rapid, high-throughput testing of a greater number of experimental animals. Consistent with our continuous recording result, subcutaneous administration of 2 mg/kg calpeptin produced an overall positive effect on ΔFRET, suggesting inhibition of calpain activity when compared to vehicle treatment. We also observed a dosage threshold, whereby lower doses of calpeptin (0.2 and 1 mg/kg) did not produce any statistically significant increases in maximum ΔFRET when compared to vehicle treatment, suggesting that our novel methodology can be used to differentiate between efficacious and non-efficacious doses of calpain inhibitor compounds.

Oral administration of 1 mg/kg calpeptin was also found to increase ΔFRET and produce a positive area under the curve, suggesting inhibition of calpains within cerebellar neurons. Surprisingly, whilst we observed a statistically significant increase in maximum ΔFRET following oral administration of 2 mg/kg calpeptin, the total area under the curve did not statistically differ from vehicle treatment. Visualization of the change in ΔFRET over the course of the 3 h following drug administration revealed that oral administration of 2 mg/kg calpeptin produced a rapid increase in FRET by 1 h post-administration, followed by a FRET signal that neared 0 (pre-administration baseline) at 2 h post-administration, falling to negative ΔFRET by 3 h post-administration, suggesting activation of calpains. This finding is interesting as treatment with calpeptin has previously been reported to downregulate levels of calpastatin, an endogenously expressed calpain inhibitor, in skeletal muscle^83^ and myoblast cultures.^84^ Thus, it is plausible that oral administration of 2 mg/kg calpeptin may initially inhibit calpains in cerebellar neurons, leading to a positive maximum ΔFRET, but this may be followed by a compensatory increase in calpain activity, perhaps due to decreased calpastatin activity. We speculate that administering high doses of exogenous calpain inhibitor compounds could trigger downregulation of calpastatin, disinhibiting calpains, whilst lower doses may not trigger this effect.

We also tested the effects of BLD-2736, a novel inhibitor of calpain 1, 2 and 9 and cathepsin K produced by Blade Therapeutics, which we had previously found to improve swimming performance in transgenic SCA3 zebrafish.^69^ We found that oral administration of various doses of BLD-2736 produced an increase in FRET signal and a positive area under the curve, suggesting that BLD-2736 crosses the BBB and inhibits calpain proteases within the brain. We similarly found that oral administration of 50 mg/kg SNJ-1945, a dose previously shown to reduce calpain expression and neuronal cell death *in vivo,*^29,77^ produced a positive and statistically significant increase in ΔFRET and a positive area under the curve when compared to vehicle treatment, indicating calpain inhibition.

Overall, we observed an equilibrium in FRET signal, whereby FRET signal stabilized from 2–3 h post-administration. This trend was consistent across all calpain inhibitor treatments, except oral administration of 2 mg/kg calpeptin. At time 0 (prior to calpain inhibitor administration), we assume there is baseline activity of calpains and that the calpain sensor construct is being readily synthesized. At 1 h post-administration, we observe a distinct increase in detected FRET signal relative to time 0, with an increase in FRET signal indicating an increased amount of intact/full-length calpain sensor, as newly synthesized calpain sensor is not cleaved due to decreased calpain activity. Interestingly, we do not observe a continued increase at 2–3 h post-administration; instead, we observed a steady equilibrium where FRET levels are similar to what was observed at 1-h post-administration. With continued synthesis of new calpain sensor protein, it could be anticipated that FRET signal would continue to increase. One reason for this stabilization in FRET signal could be reduced calpain inhibitor effectiveness at 2–3 h post-administration compared to 1-h post-administration. It is possible that the transient effects of administered calpain inhibitor compounds may be beginning to wear off, leading to a slow reactivation of calpain activity and consequent cleavage of the calpain sensor (similar to what was observed at 3-h post-administration of 2 mg/kg calpeptin). We hypothesize that slow reactivation of calpains as drug effects wane could lead to an equilibrium and steady FRET signal; however, further pharmacological studies may be required to investigate this effect further. An alternative interpretation is that protein synthesis of the calpain sensor construct could also be reduced with inhibition of calpain activity, meaning that whilst cleavage of the calpain sensor is reduced, protein synthesis is also reduced, leading to a steady FRET signal.

Finally, administration of ionomycin, an ionophorus antibiotic that acts to transport stored calcium ions into the cytosol^79^ and is known to increase calcium concentrations within cerebellar neurons,^85^ produced a negative change in FRET signal when compared to pre-treatment levels that was statistically significant. This highlights that our novel methodology could also be used to investigate drugs known to increase calcium signalling or calpain activity in cerebellar neurons.

We observed a higher maximum ΔFRET value following intraperitoneal administration of DMSO than administration of other vehicles (orally administered 0.5% methyl cellulose and subcutaneous 0.9% saline); however, comparison of all three vehicles did not reveal a statistically significant difference. We hypothesize that the slight, albeit non-significant, elevation in maximum ΔFRET produced by DMSO is unlikely to be caused by inhibition of calpains, but more likely caused by an effect on the calpain sensor fluorescence emission. A steady-state fluorescence spectroscopy study by Markarian and Shahinyan^86^ revealed that application of DMSO to water containing the stain acridine orange led to a dose-dependent increase in fluorescent signal between 450 and 600 nm, with higher doses of DMSO causing greater increases in fluorescent intensity. Whilst the parameters of our study vastly differ from that of Markarian and Shahinyan, it is plausible that administration of DMSO may enhance fluorescent signal between 450 and 600 nm, matching the wavelengths of light that pass through our band-pass dichroics in our fibre photometry system. It is plausible that the slight elevation in FRET signal observed following injection of DMSO may be due to the effects of DMSO on fluorescence intensity. Considering this, future experiments employing the novel methodology described in this paper or other fluorescent-based methodologies developed to measure drug penetrance across the BBB may want to avoid DMSO as a solvent due to possible confounding artefacts.

### Advantages of our approach and implications for the calpain field

To our knowledge, we are the first to develop a method of detecting changes in neuronal calpain activity *in vivo*. We have highlighted that this methodology can be used for preclinical testing of compounds predicted to modulate calpain activity. The calpain sensor construct used in this study is not specific to one isoform of calpain. This is a disadvantage for experiments aiming to elucidate isoform-specific activity information, but it is advantageous when comparing the efficacy of a broad range of calpain inhibitor compounds that target different isoforms of calpains. This methodology can be used as a correlate of brain penetrance of novel drugs and would be especially informative in conjunction with pharmacokinetic measures to characterize the relationship between pharmacokinetics and pharmacodynamics, which is crucial to translational drug development. Further, our novel approach of measuring neuronal calpain activity within neurons in awake mice could be combined with behavioural tasks or physiological manipulations, providing new insight into the calpain field.

To date, most experimental work utilizing FRET-based calpain sensors *in vivo* has explored activity of calpains within skeletal muscle.^66,72,87^ Use of CAFI mice, which ubiquitously express a calpain sensor in many tissues, including the brain, is another alternative method for exploring neuronal calpain activity *in vivo*.^72^ Our approach, utilizing an AAV to drive expression of the calpain sensor, offers a more cost-effective approach to examining calpain activity in transgenic disease models, bypassing the need to breed and cross multiple transgenic lines. However, implementing our fibre photometry approach in CAFI mice could offer advantages over AAV-calpain sensor delivery, as there would be more ubiquitous expression of the calpain sensor throughout the brain, mitigating the risk of off-target fibre implantation and potentially reducing variability in calpain sensor expression across experimental animals.

Our study also pairs the calpain sensor, a useful tool to monitor and record calpain activity, with the use of fibre photometry, enabling examination of neuronal calpain activity in awake mice The ability to record real-time changes in calpain activity in behaving mice may be beneficial to researchers hoping to explore changes in calpain activity during certain behavioural tasks or physiological states (e.g. sleep, fasting, pregnancy, aging). Further, our approach enables investigation of calpain activity without any potential death-induced changes in calpain activity confounded by death^65^ and allows repeated testing of calpain-inhibiting compounds within the same experimental animal, meaning each experimental animal acts as its own vehicle control, reducing the economic and ethical cost of research.

In this study, we investigated changes in calpain activity within neurons of the cerebellum, as previous work from our laboratory suggested that calpain activity may be increased in the cerebellum of CMVMJD135 mice modelling SCA3. Future studies could utilize the methodology described here to investigate real-time changes in neuronal calpain activity in other areas of the brain, such as the hippocampus, where the role of respective calpain isoforms has been more extensively studied.

### Limitations and caveats

Our novel methodology also faced some challenges and caveats. Firstly, we found that we were no longer able to detect a clear FRET signal ∼4–5 months after instrumentation, although reporter-expressing neurons could still be clearly identified under post-mortem histological examination. Our correlation analysis revealed that the number of days between viral transduction surgery and euthanasia was correlated with the number of calpain sensor-expressing neurons at euthanasia (as detected via microscopy). This time variable was independent to the number of fibre photometry test sessions experimental animals were exposed to. These findings suggest that the loss of calpain sensor expression cannot be attributed to fibre photometry or photobleaching alone but could be associated with time-dependent factors. AAV-mediated expression is relatively stable in non-mitotic neurons, and our ability to detect calpain sensor expression via microscopy suggests some level of calpain sensor expression was maintained. The loss of detectable FRET signal was not random; animals that maintained a FRET signal detectable by fibre photometry at euthanasia were found to express the calpain sensor construct in >200 neurons. In contrast, in experimental animals where FRET could no longer be detected by fibre photometry, <200 calpain sensor expressing neurons were observed. Thus, it is possible fibre photometry is limited in detecting FRET signals when lower numbers of neurons (i.e. < 200) are expressing the calpain sensor. Interestingly, Bartoli and colleagues^72^ have reported a loss of FRET signal in skeletal muscle in 4-week-old CAFI mice, which transgenically express calpain sensor. Bartoli and colleagues postulated that the loss of calpain sensor expression could be due to the death of calpain-sensor-expressing cells.^72^ Considering our mice were reaching 6–7 months of age at the time of signal loss, it is plausible that cell death may be occurring in the cerebellum, leading to a reduction in FRET-expressing neurons. It is also possible that our inability to detect a FRET signal at late stages of the experiment could also be due to blockage of or damage to implanted fibre optic cannulae. Future studies hoping to employ this methodology should be aware of cell death mechanisms that may lead to time-dependent decay of calpain sensor expression and should balance testing of many compounds, doses or administration routes, which could increase the experimental timeframe and risk calpain sensor expression decaying below a detectable threshold in long experiments, against testing a shorter number of compounds/doses in a shorter timeframe when calpain sensor expression may be stronger and less variable.

Additionally, over time we found that mice were prone to scratching at the dental cement used to secure fibre optic implants. This, combined with the stress placed on implants during treatment administration via oral gavage and fibre attachment, led to accidental removal of the implanted fibre optic cannula in several experimental animals, resulting in incomplete within-animal testing of all compounds, administration routes, doses and vehicles before loss of FRET signal or implanted fibre. This diminished the power of our analysis, as we were not able to conduct repeated measures statistical analysis and required more experimental animals than first planned to complete the proposed testing.

Unlike traditional fibre photometry approaches using calcium and neurotransmitter sensors, we could not employ the use of an isosbestic channel as an activity-independent control, as the isosbestic point of the FRET-EYFP sensor is not well characterized. Therefore, for an activity-independent control, we measured fluorescence from the brain while the excitation LED was off. This was likely sufficient to control for motion artefacts (e.g. fibre bending, scratching), and potentially for changes in brain autofluorescence over the recording session. However, as our control signal did not involve fluorescent excitation (unlike the isosbestic controls used for calcium sensors), we could not measure the extent of LED-induced photobleaching, which likely would have occurred throughout the recordings. To that end, we attempted to mitigate the extent of photobleaching by stimulating for only brief periods of time (1–2 min every 60 min). Future use of our novel method should incorporate improved photobleaching controls, such as co-expression of a red-shifted activity-independent fluorophore (e.g. tdTomato, as used here^88^), which can be spectrally or temporally resolved from the FRET-EYFP signal. It should be noted that insufficiently controlling for photobleaching would presumably underestimate the magnitude of the increased fluorescent signal in response to calpain inhibitors demonstrated here. Overall, this limitation does not impair our ability to draw conclusions about the novel compounds tested, yet improving photobleaching controls would improve sensitivity to less efficacious or potent drugs and calpain manipulations.

Our *in silico* analysis revealed that the calpain sensor construct is likely to be cleaved by both calpain 1 and calpain 2, with similar prediction scores for both calpain isoforms. Thus, the use of this particular calpain sensor does not allow for the identification of which calpain isoform is active. Future studies could employ our fibre photometry approach with a different FRET-based sensor that can distinguish between calpain isoforms, providing new valuable insights into the relative activity of different calpain isoforms following administration of different calpain inhibitors.

Lastly, we have observed high variability within our data, with individual responses to drug effects varying across experimental animals. Factors such as variation in viral expression and proximity of implanted fibre optic cannulae to viral expression, time-mediated decay of viral expression, genotype differences in basal calpain activity and individual responsiveness to administered compounds could explain the differences in observed FRET signal, meaning that this approach may be useful for looking at changes in FRET activity over time within the same experimental subject but is not suitable for examining absolute changes in calpain activity between individual animals. Further, in our hands, transgenic SCA3 mice recovered poorly from surgery compared to wildtype controls: researchers wishing to utilize this methodology in other disease models should note that recovery from the surgical procedure may be challenging in diseased animals. Future experiments could utilize transgenic CAFI mice, described by Bartoli *et al.*,^72^ to control for factors such as variation in viral transduction or misalignment of implanted fibre optrode.

## Conclusion

In the present study, we developed a novel methodology combining viral expression of a FRET-based calpain sensor and implantation of a fibre optic cannula, facilitating real-time recording of calpain activity within cerebellar neurons in awake mice. To our knowledge, this is the first reported study to examine calpain activity within neurons of the central nervous system *in vivo*. Our results confirm that this methodology can be used to examine changes in FRET signal, a proxy for calpain activity, following administration of compounds that inhibit or activate calpain proteases. Our repeated within-animal approach also facilitated direct comparison of different administration routes and doses, allowing us to identify the most efficacious treatment regime for inhibiting calpains within cerebellar neurons. Our methodology may increase the speed and validity of compound testing and lead to better optimization of treatment strategies, enhancing preclinical drug testing pipelines and potentially leading to more successful translation to clinical populations.

## Supplementary Material

fcag150_Supplementary_Data

## Data Availability

Data relating to this study are available from the corresponding author on reasonable request. The Bonsai workflow used in the photometry experiment is available at github.com/angelaird-neuro/photometry_bonsai.
