# Supplementary material for: Using fibre photometry with a fluorescence resonance energy transfer-based biosensor to test efficacy of calpain inhibitors in vivo
Source: Brain Commun. 2026 Apr 24;8(3):fcag150. doi: 10.1093/braincomms/fcag150 (PMC13178110; doi:10.1093/braincomms/fcag150)
Supplement: fcag150_Supplementary_Data [file fcag150_supplementary_data.pdf]

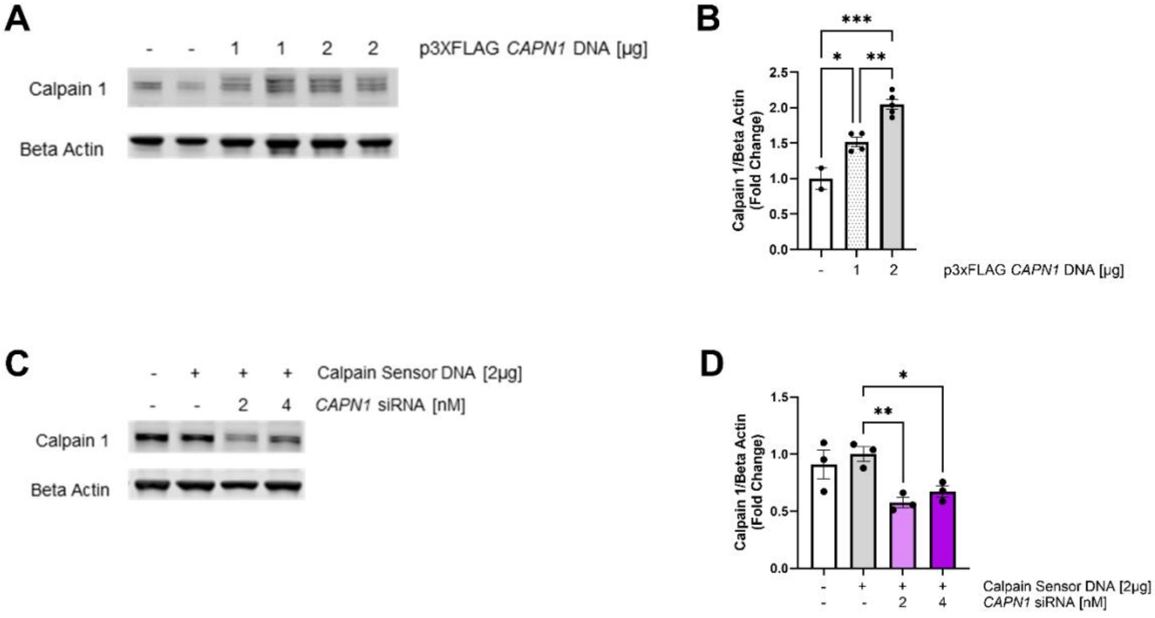

Supplementary Figure 1 Effect of genetic manipulation of *CAPN1* on calpain 1 protein levels. (**A**) Representative western blot image of NSC-34 cells overexpressing calpain 1 (p3xFLAG *CAPN1*). (**B**) Quantitative analysis of calpain 1 protein levels following calpain 1 expression. (**C**) Representative western blot of calpain 1 abundance in untransfected NSC-34 cells, NSC-34 cells expressing calpain sensor and NSC-34 cells expressing calpain sensor and *CAPN1* siRNA (2 or 4 nM). (**D**) Quantitative analysis of the amount of calpain 1 in cells expressing calpain sensor construct and *CAPN1* siRNA. Data was analysed using one-way ANOVA with post-hoc multiple comparisons. **(B)** and **(D)** display the group mean and SEM. * represents $P < 0.05$ and ** represents $P < 0.01$ and *** represents $P < 0.001$. Each dot point represents immunoblot band density in a single experimental replicate (n = 3-6 per group).

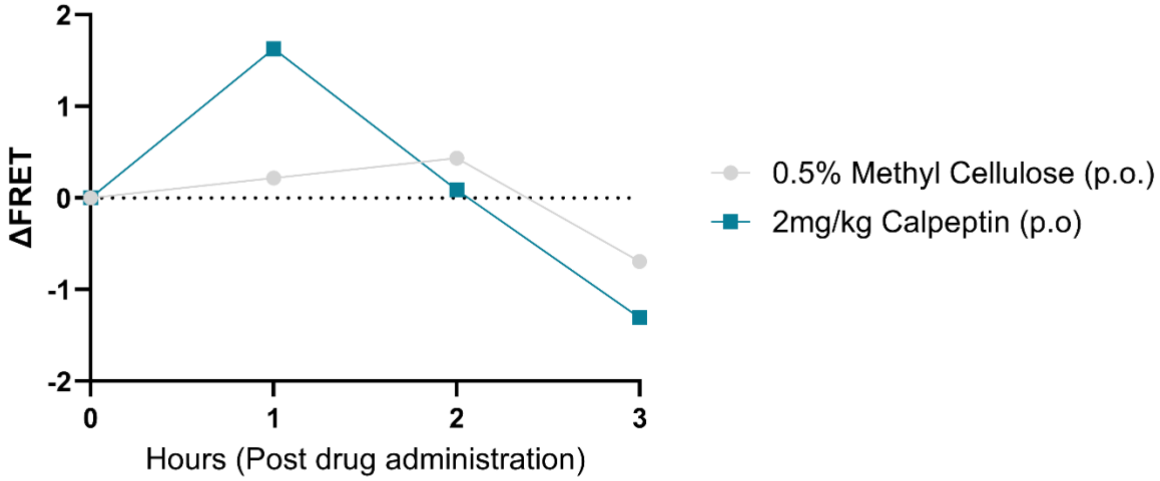

Supplementary Figure 2 Representative graph displaying the changes in ΔFRET following oral administration of 2mg/kg calpeptin. Each trace represents the change in FRET signal following administration of 2mg/kg calpeptin (teal) or vehicle (grey) in the same experimental animal (n = 1). Data presented within this figure was not statistically analysed.

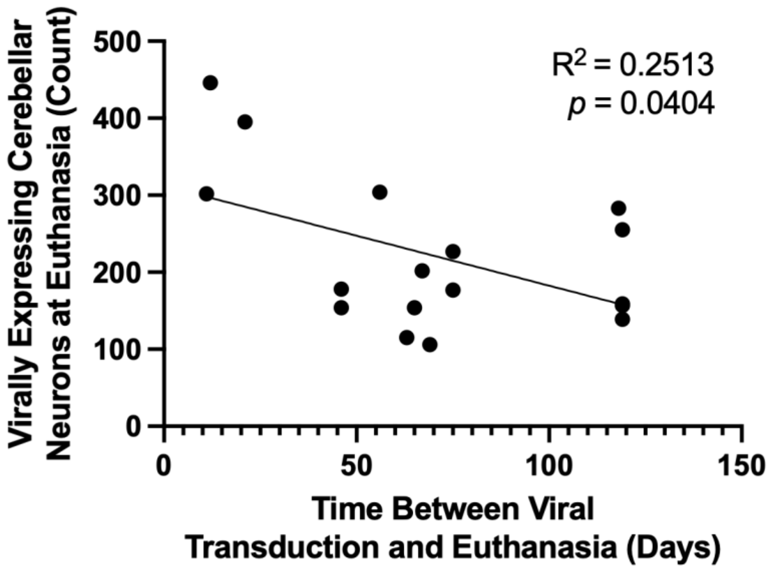

Supplementary Figure 3 Analysis of the relationship between the number of virally transduced neurons at euthanasia and the number of days between viral transduction

surgery and euthanasia. Data was statistically analysed using Pearson's Correlation Coefficients. Each dot point represents a single experimental animal (*n* = 16).

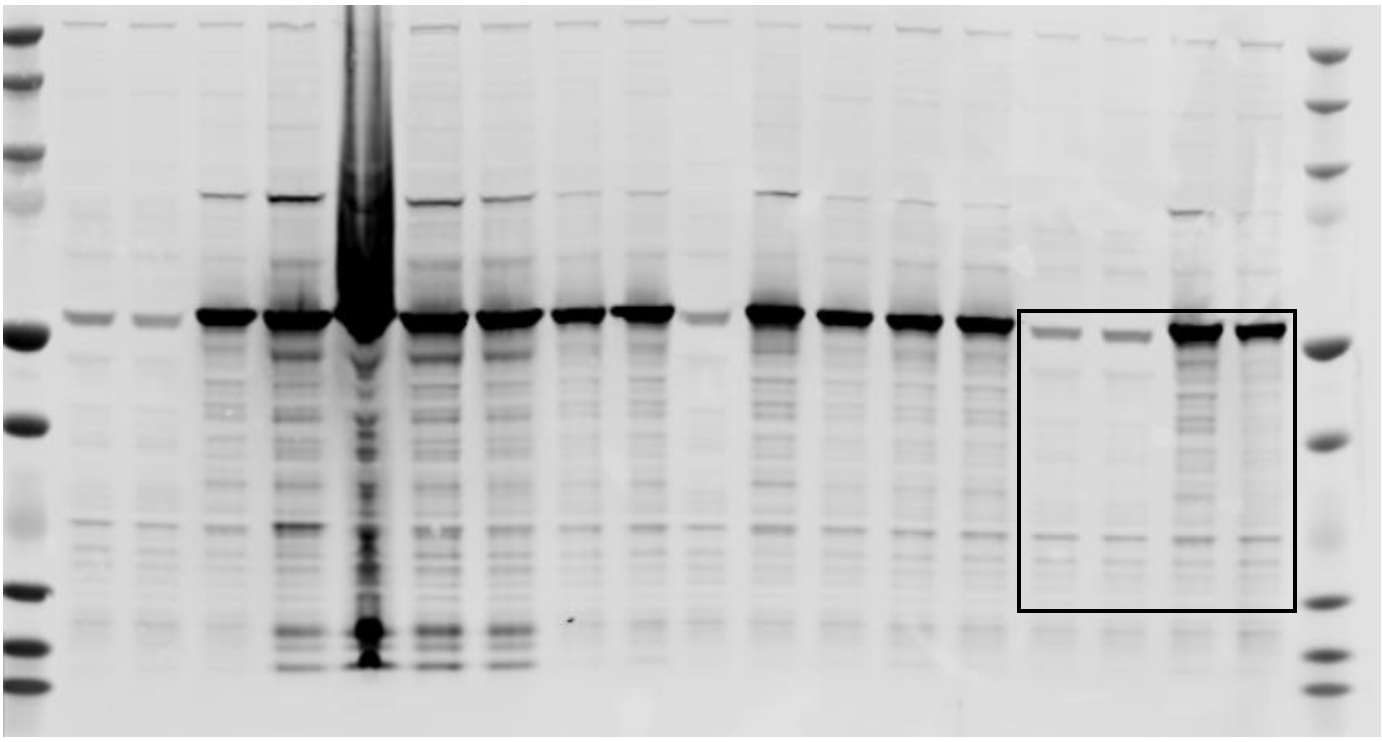

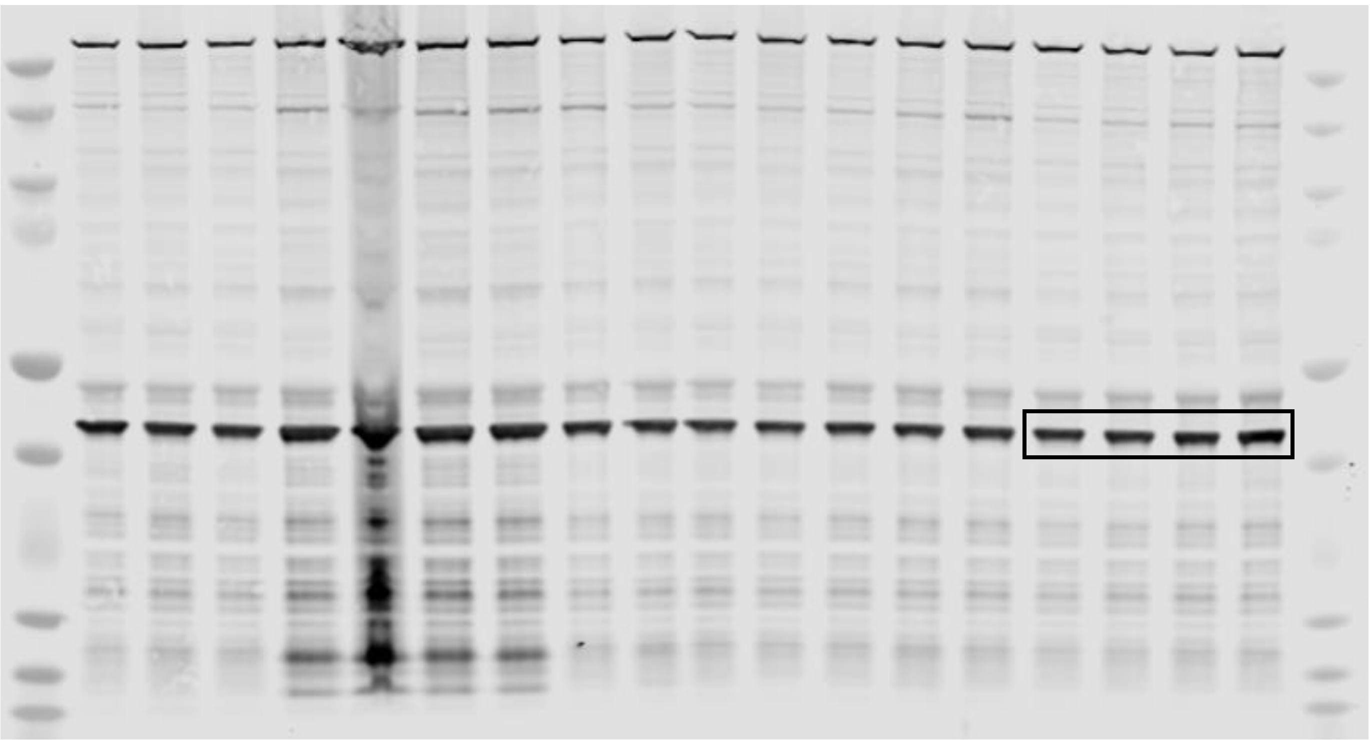

Full GFP blot and matching beta actin blot shown in Figure 3A.

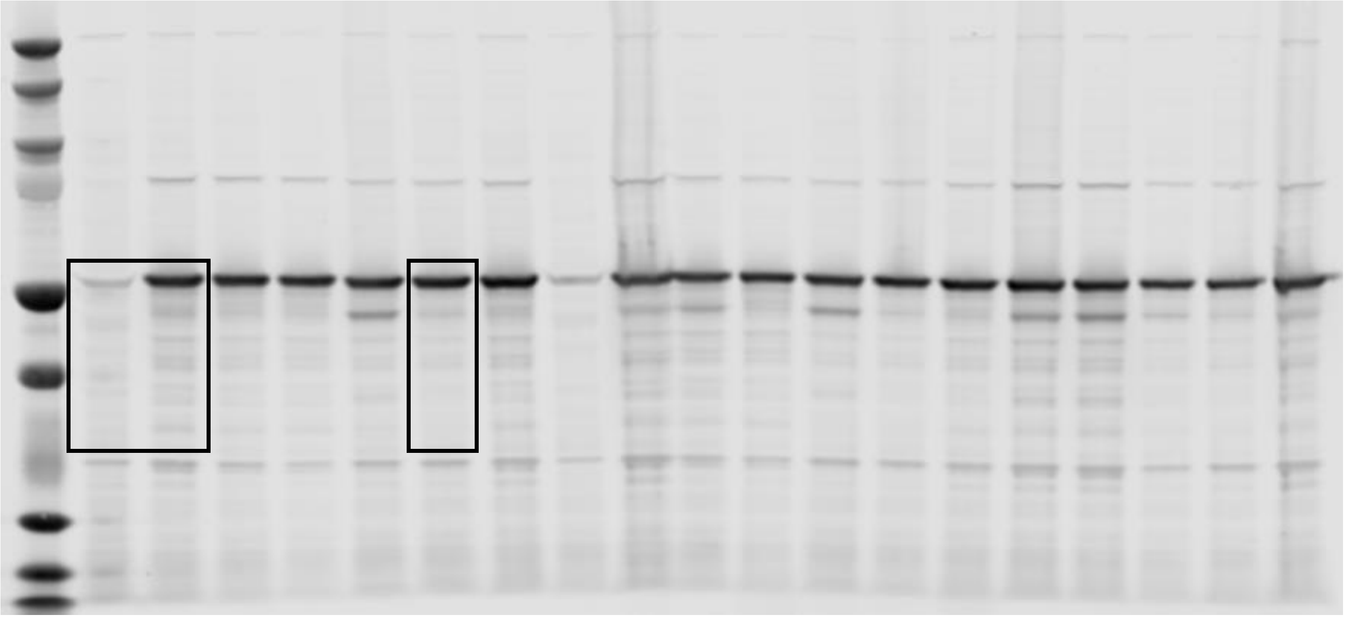

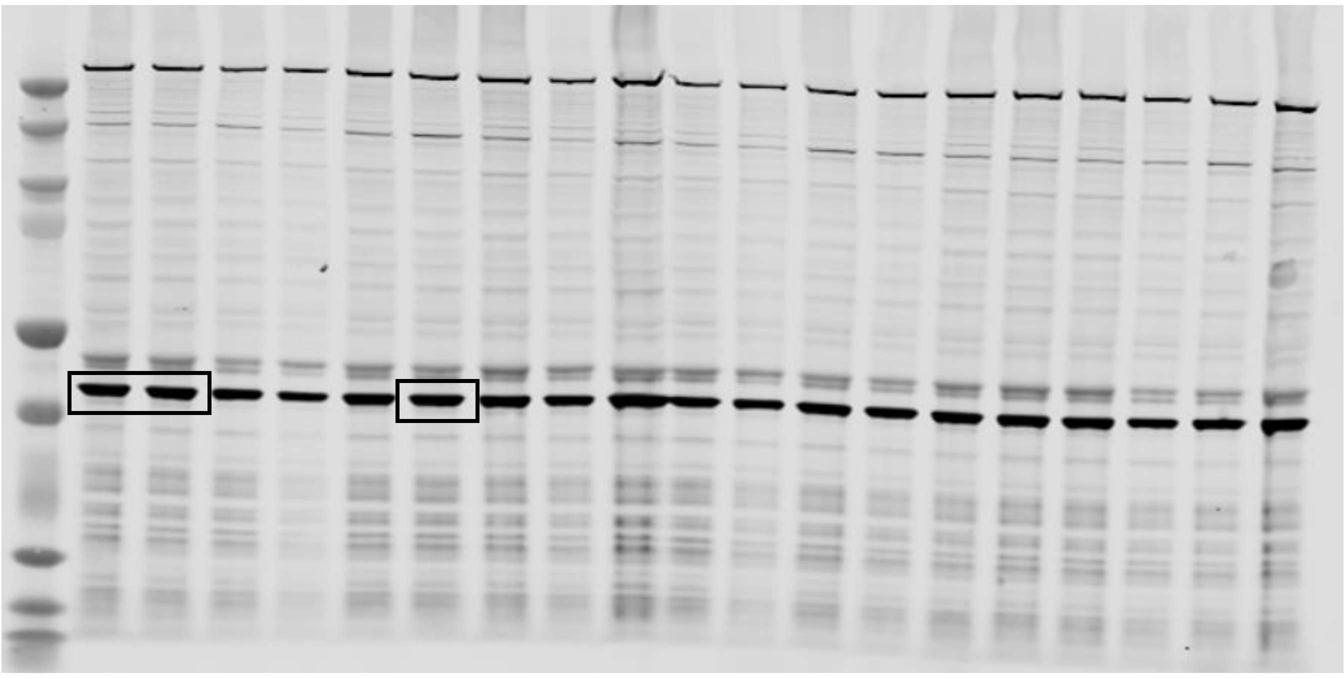

Full GFP blot and matching beta actin blot shown in Figure 3C.

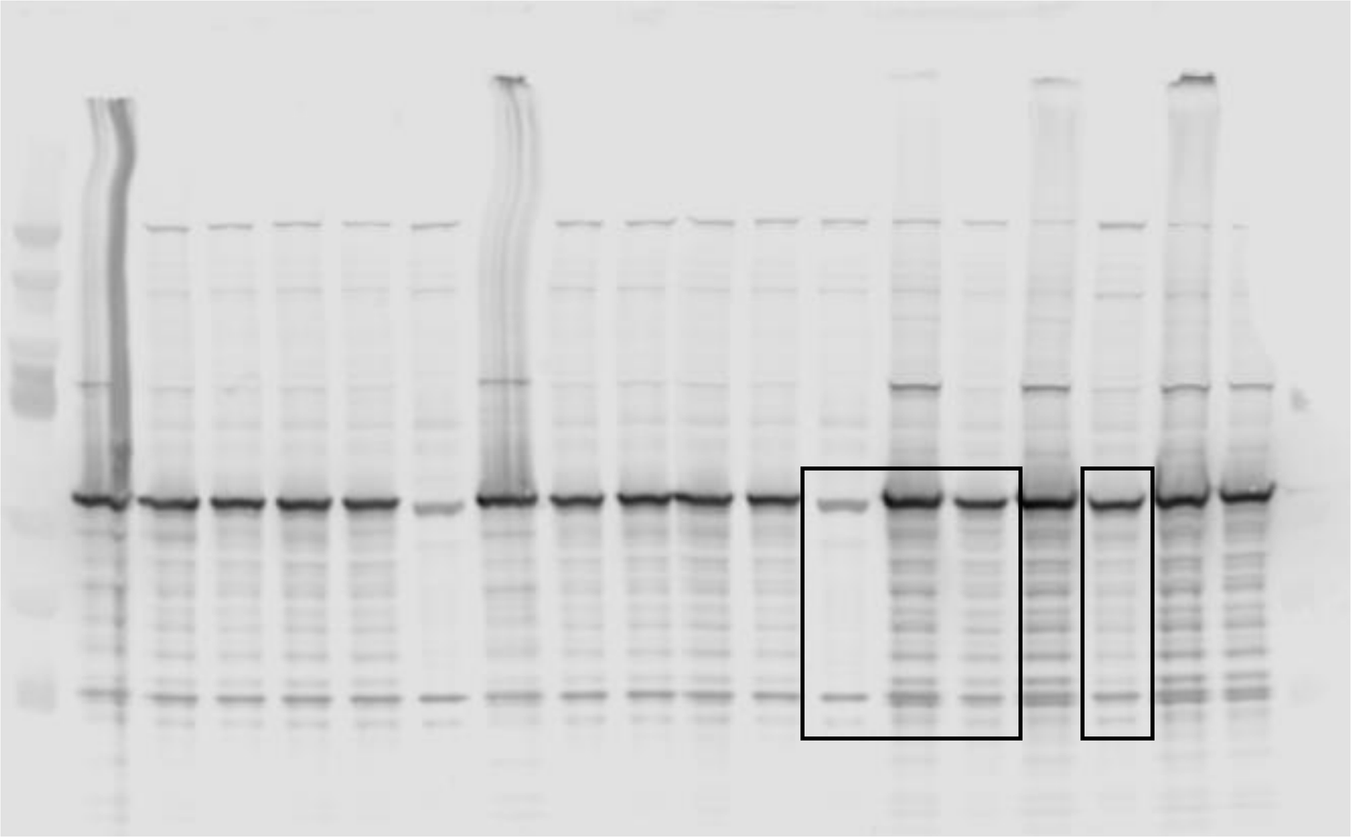

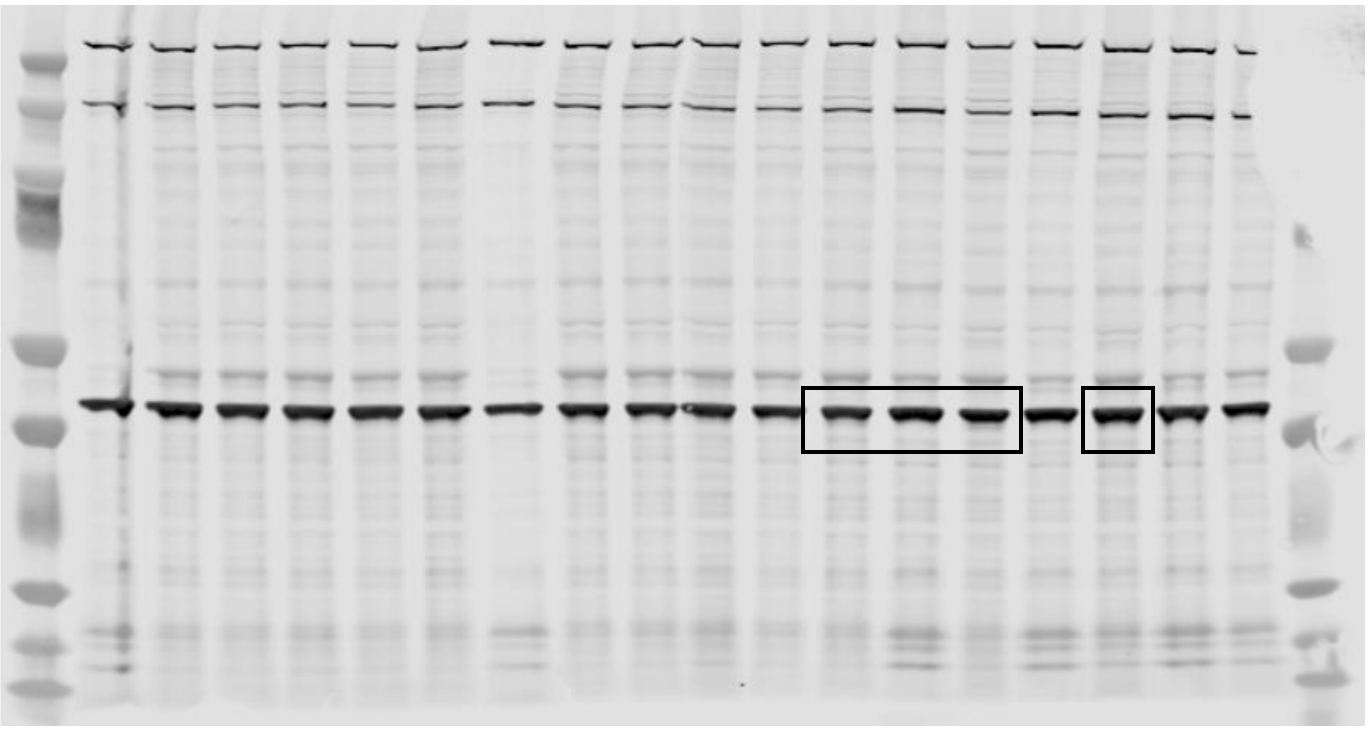

Full GFP blot and matching beta actin blot shown in Figure 3E.

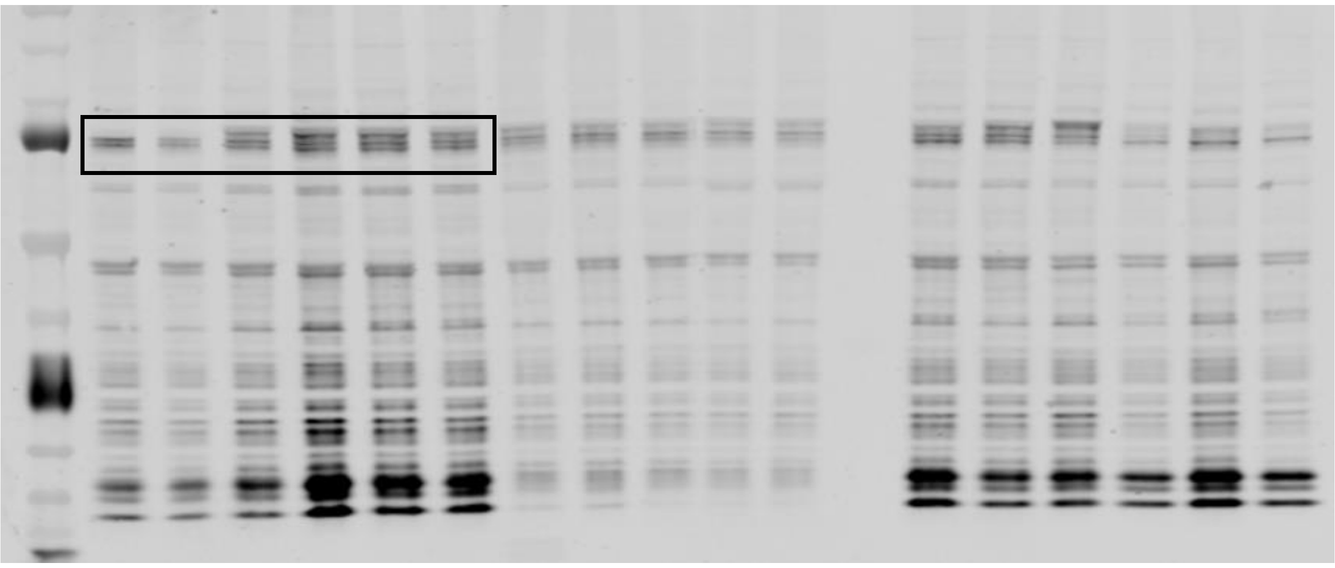

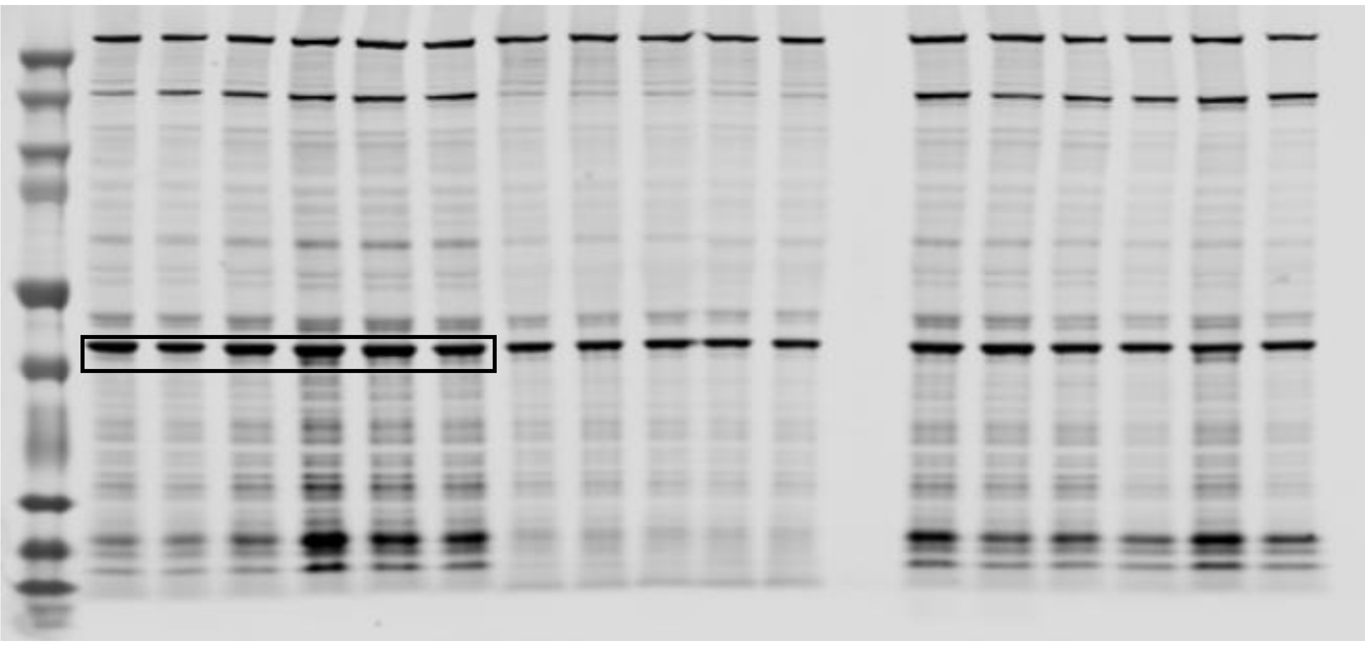

Full calpain 1 blot and matching beta actin westerns shown in Supplementary Figure 1A.

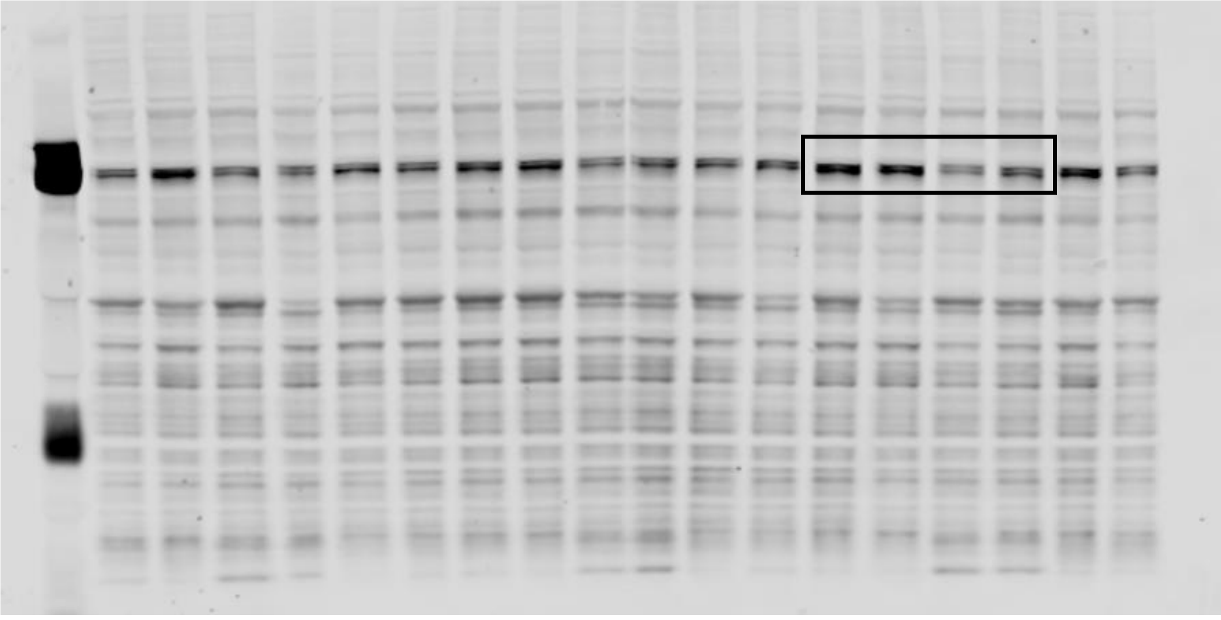

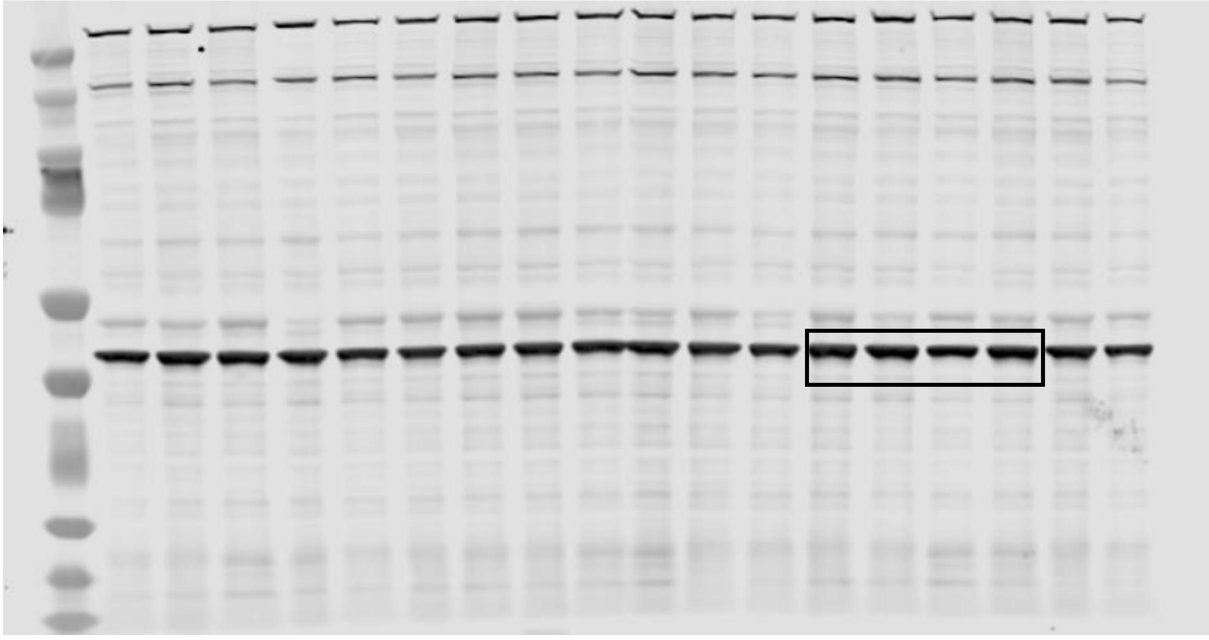

Full calpain 1 siRNA blot and matching beta actin westerns shown in Supplementary Figure 1C.
